# Species groups and identification key to Chinese *Temnothorax* ants (Hymenoptera, Formicidae) with descriptions of four new species from Shennongjia National Park

**DOI:** 10.3897/zookeys.1292.180703

**Published:** 2026-09-18

**Authors:** Fangchao Deng, Jingyuan Yang, Zhenghui Xu

**Affiliations:** 1 Key Laboratory of Forest Disaster Warning and Control in Yunnan Province / College of Forestry, Southwest Forestry University, Kunming, Yunnan Province 650224, China Key Laboratory of Forest Disaster Warning and Control in Yunnan Province / College of Forestry, Southwest Forestry University Kunming China; 2 Shennongjia National Park Administration, Muyu, Hubei Province 442421, China Shennongjia National Park Administration Muyu China

**Keywords:** Checklist, key, Myrmicinae, new species, species groups, *
Temnothorax
*

## Abstract

The myrmicine ant genus *Temnothorax* Mayr, 1861 is highly diverse in China; however, its fauna remained inadequately studied until recently. During the 2023 Background Resources Survey Project in Shennongjia National Park, four new species of this genus were discovered and are herein described as *Temnothorax shennongi***sp. nov**., *T. hubeiensis***sp. nov**., *T. zhaojunae***sp. nov**., and *T. wangweii***sp. nov**. To date, a total of 66 species of *Temnothorax* have been documented in China. To facilitate precise identification and differentiation of these species, a checklist of all known Chinese species of the genus was compiled, sorted into 20 species groups, and a diagnosis for each group is provided. Additionally, a revised identification key for the worker caste of the 66 species based on the species groups is presented. This study not only increases the number of known *Temnothorax* species in China but also systematizes the taxonomic knowledge of the genus.

## Introduction

The myrmicine genus *Temnothorax* was established by Mayr (1861). Subsequently, it was regarded by various authors as a sub-genus of *Leptothorax* Mayr, 1855 (Forel 1892; Ruzsky 1905; Wheeler 1910, 1922; Emery 1915, 1924; Bondroit 1918; Donisthorpe 1943) or as a junior synonym of *Leptothorax* (Forel 1890; Baroni Urbani 1971; Brown Jr 1973; Bolton 1994; Terayama and Onoyama 1999). More recently, *Temnothorax* has been re-established and is widely recognized as a valid genus (Bolton 2003; Radchenko 2004). In addition to morphological evidence, molecular phylogenetic studies have provided further support for the separation of *Temnothorax* from *Leptothorax* (Ward et al. 2015; Prebus 2017).

*Temnothorax* Mayr, 1861 is a genus of substantial size and wide distribution, spanning the Palearctic, Oriental, Afrotropical, Nearctic, and Neotropical regions (Janicki et al. 2016). To date, 505 species and 29 subspecies of the genus are known worldwide, with the majority recorded from Europe, North Africa, and North America (Bolton 2025). Although numerous myrmecologists have contributed to the study of *Temnothorax*, comprehensive systematic revisions of the Chinese fauna remain relatively scarce. Previous works by Radchenko (2004), Terayama (2009), and Zhou et al. (2010) revised the eastern Palearctic and Oriental representatives of the genus; however, more extensive and in-depth investigations on the Chinese fauna are still needed. More recently, Hamer et al. (2023) described two new species from Hong Kong, China, namely *T. barrettoi* Hamer & Guénard and *T. haveni* Lee et al. In the subsequent year, Qian and Xu (2024) synonymized *Temnothorax opaciabdomin* (Chang & He, 2001) with *T. mongolicus* (Pisarski, 1969) and described 28 new species from China. These findings indicate that the diversity of *Temnothorax* in China remains insufficiently understood and that the taxonomy of the genus in this region requires further clarification.

In the present study, we document four new species of *Temnothorax* discovered during the 2023 Background Resources Survey Project in Shennongjia National Park, Hubei Province, which lasted 26 days. The new species inhabit semi-evergreen broadleaf forest and conifer-broadleaf mixed forests at altitudes of 550–1700 m. They are notably rare: among the 3537 ant individuals collected, representing four subfamilies, 26 genera, and 68 species, only nine individuals belonged to the four new species (*T. hubeiensis* sp. nov., four individuals; *T. shennongi* sp. nov., two; *T. wangweii* sp. nov., one; and *T. zhaojunae* sp. nov., two). Similar rarity patterns were observed in the work of Qian and Xu (2024). Based on our surveys of ant communities and diversity in western China during the past three decades, we found that species of the *Temnothorax* are characterized by a small body size and small populations. Therefore, its individuals are inconspicuous and rarely captured. Among all the species we collected, only a few were common, while the majority were rare. Given China’s vast territory, it is urgent to investigate and record different ant species in today’s rapidly changing environment. Under the conditions of limited funding for taxonomic research, it is difficult to conduct repeated surveys on the ant fauna of a region. Therefore, relying on limited individual advancement for classification description has become an inevitable choice at present. The four new species are described as part of a broader revision of the Chinese *Temnothorax* fauna, addressing rare taxa. Shennongjia National Park, located at the border mountains of Hunan, Guizhou, Sichuan and Hubei, is one of China’s 11 key terrestrial biodiversity conservation regions. Despite its ecological importance, the park is in central China, surrounded by dense human populations and subject to anthropogenic disturbances. This study increases the known Chinese *Temnothorax* fauna from 62 to 66. To facilitate precise identification and distinction among these complex taxa, we prepared a checklist of all 66 species, divided them into 20 species groups, and provided diagnostic characteristics for each group. In addition, a revised identification key for all known Chinese species of the genus is constructed based on the species grouping.

## Materials and methods

Chinese *Temnothorax* species were studied based on specimens collected from China as well as type and non-type specimen images accessible on the AntWeb (http://www.antweb.org) and AntWiki (http://www.antwiki.org). The foreign type specimens were not personally inspected but taxonomic conclusions were founded on high-resolution photographs of type material. For four species, *T. argentipes* (Wheeler, 1928), *T. eburneipes* (Wheeler, 1927), *T. taivanensis* (Wheeler, 1929a), and *T. wui* (Wheeler, 1929b), color images were unavailable; thus, the original descriptions and line drawing illustrations provided by Radchenko (2004) were utilized for comparison and species identification. Ant specimens from Shennongjia National Park, Hubei Province, China, were collected through searching method described by Xu et al. (2011). *Temnothorax* species were examined under a SDPTOP SZ stereo-microscope with a micrometer (Sunny Optical Technology (Group) Co. Ltd, Zhejiang, China). Digital images were obtained using a Liyang Super Resolution System LY-WN-YH (Chengdu Liyang Precision Machinery Co., Ltd, China), and image stacking was performed with Zerene Stacker software (Zerene Systems LLC, USA). Morphological terminology and integument sculpture characters conformed to the glossary provided in Hölldobler and Wilson (1990) and Bolton (1994). Unique Specimen Identities (USIs) of specimens housed in the Ant Collection of Southwest Forestry University (**SWFU**) are incorporated in the “Type Material” section of each new species description to facilitate repeatability enabling future researchers to reference the material. The ant collection catalog follows the format “SWFU A23-1”, where SWFU is the institution abbreviation, A (or B, C) denotes the person responsible for the specimens, 23 corresponds to the last two digits of the collection year (2023), and 1 is the starting sequential number of the ant specimens collected in the year 2023. Multiple individuals from the same nest were assigned a single specimen number to indicate their nest origin. All the type specimens of the four new species are deposited in the Ant Collection of Southwest Forestry University (**SWFU**), Kunming, Yunnan Province, China, except one paratype worker (SWFU A23-906) which will be donated to the Insect Collection, Guangxi Normal University, Guilin, Guangxi Zhuang Autonomous Region, China.

Standard measurements and indices adhere to the methodology proposed by Bolton (1983), with the inclusion of ED, PL, PH, and DPW as delineated hereinafter. All measurements are expressed in millimeters (mm).

**TL** Total Length: total outstretched length of the individual, from the mandibular apex to the gastral apex.

**HL** Head Length: straight-line length of head in full-face view, measured from the mid-point of the anterior clypeal margin to the midpoint of the posterior margin. For species with concave margins, the measurement is taken from the mid-point of a transverse line connecting the apices of the projecting portions.

**HW** Head Width: maximum width of head in full-face view, excluding the eyes.

**SL** Scape Length: straight-line length of the antennal scape, excluding the basal constriction or neck.

**ED** Eye Diameter: maximum diameter of the eye.

**PW** Pronotal Width: maximum width of pronotum in dorsal view.

**WL** Weber’s length: diagonal length of the mesosoma in profile from the point where pronotum meets the cervical shield to the posterior base of the metapleuron.

**PL** Petiole Length: length of petiole measured in lateral view, from the anterior to posterior articulation.

**PH** Petiole Height: height of petiole in lateral view from the apex of the ventral (subpetiolar) process vertically to a line intersecting the dorsal most point of the node.

**DPW** Dorsal Petiole Width: maximum width of petiole in dorsal view.

**CI** Cephalic Index = HW × 100 / HL.

**SI** Scape Index = SL × 100 / HW.

### Acronyms of specimen repositories

**CAF** Organism Specimen Collection, Institute of Forest Ecological Environment and Nature Conservation, Chinese Academy of Forestry, Beijing, China.

**GXNU** Insect Collection, Guangxi Normal University, Guilin, Guangxi Zhuang Autonomous Region, China.

**MCZC** Museum of Comparative Zoology, Harvard University, Cambridge, Massachusetts, USA.

**SWFU** Ant Collection, Southwest Forestry University, Kunming, Yunnan Province, China.

## Taxonomic account

### List of known Chinese *Temnothorax* species with geographical distribution

To date, 66 species of *Temnothorax* are recognized from China, including the four new species described in this study. The currently known Chinese species are listed below along with their recorded distributions.

1. *T. angulohumerus* Zhou et al., 2010, Palearctic and Oriental Regions: China: Guangxi, Guizhou, Hunan, Xizang, Yunnan.
2. *T. argentipes* (Wheeler, 1928), Palearctic and Oriental Regions: China: Beijing, Fujian, Guangxi, Hebei, Henan, Jiangxi, Liaoning, Ningxia, Shaanxi, Sichuan, Yunnan.
3. *T. bailu* Qian & Xu, 2024, Palearctic and Oriental Regions: China: Sichuan, Xizang, Yunnan.
4. *T. barrettoi* Hamer & Guénard, 2023, Oriental Region: China: Hong Kong.
5. *T. brevispinus* (Chang & He, 2001), Palearctic Region: China: Ningxia, Qinghai.
6. *T. chun* Qian & Xu, 2024, Oriental Region: China: Yunnan.
7. *T. chunfen* Qian & Xu, 2024, Oriental Region: China: Yunnan.
8. *T. chushu* Qian & Xu, 2024, Oriental Region: China: Sichuan, Yunnan.
9. *T. confucii* (Forel, 1912), Oriental Region: China: Taiwan.
10. *T. congruus* (Smith, 1874), Palearctic and Oriental Regions: China: Beijing, Guangxi, Yunnan; Japan, North Korea, South Korea, Russia.
11. *T. dahan* Qian & Xu, 2024, Oriental Region: China: Yunnan.
12. *T. dashu* Qian & Xu, 2024, Palearctic and Oriental Region: China: Xizang, Yunnan.
13. *T. daxue* Qian & Xu, 2024, Oriental Region: China: Yunnan.
14. *T. desioi* (Menozzi, 1939), Palearctic and Oriental Regions: China: Xizang; India (type locality), Pakistan.
15. *T. dong* Qian & Xu, 2024, Oriental Region: China: Yunnan.
16. *T. dongzhi* Qian & Xu, 2024, Oriental Region: China: Sichuan.
17. *T. eburneipes* (Wheeler, 1927), Palearctic and Oriental Regions: China: Jiangxi; North Korea.
18. *T. guyu* Qian & Xu, 2024, Oriental Region: China: Sichuan and Yunnan.
19. *T. hanlu* Qian & Xu, 2024, Oriental Region: China: Yunnan.
20. *T. haveni* Lee et al., 2023, Oriental Region: China: Hong Kong.
21. *T. hengshanensis* (Huang et al., 2004), Oriental Region: China: Hunan, Yunnan.
22. *T. huatuo* Terayama, 2009, Oriental Region: China: Taiwan.
23. *T. hubeiensis* sp. nov., Oriental Region: China: Hubei.
24. *T. jingzhe* Qian & Xu, 2024, Oriental Region: China: Yunnan.
25. *T. koreanus* (Teranishi, 1940), Palearctic Region: China: Hubei; North Korea, Japan, South Korea.
26. *T. kuixing* Terayama, 2009, Oriental Region: China: Taiwan.
27. *T. leigong* Terayama, 2009, Oriental Region: China: Taiwan.
28. *T. leimu* Terayama, 2009, Oriental Region: China: Taiwan.
29. *T. leyeensis* Zhou et al., 2010, Oriental Region: China: Guangxi.
30. *T. lichun* Qian & Xu, 2024, Oriental Region: China: Yunnan.
31. *T. lidong* Qian & Xu, 2024, Oriental Region: China: Sichuan, Yunnan.
32. *T. liqiu* Qian & Xu, 2024, Oriental Region: China: Sichuan, Yunnan.
33. *T. lixia* Qian & Xu, 2024, Oriental Region: China: Sichuan.
34. *T. mangzhong* Qian & Xu, 2024, Oriental Region: China: Yunnan.
35. *T. maoerensis* Zhou et al., 2010, Oriental Region: China: Guangxi.
36. *T. mongolicus* (Pisarski, 1969), Palearctic Region: China: Hebei, Inner Mongolia; North Korea, Mongolia, Russia.
37. *T. orchidus* Zhou et al., 2010, Oriental Region: China: Guangxi, Sichuan and Yunnan.
38. *T. pisarskii* Radchenko, 2004, Palearctic Region: China: Hebei, Heilongjiang, Liaoning, Shaanxi; North Korea.
39. *T. qingming* Qian & Xu, 2024, Palearctic Region: China: Xizang.
40. *T. qiu* Qian & Xu, 2024, Oriental Region: China: Sichuan, Yunnan.
41. *T. qiufen* Qian & Xu, 2024, Palearctic and Oriental Regions: China: Sichuan, Qinghai.
42. *T. reduncus* (Wang & Wu, 1988), Palearctic and Oriental Regions: China: Fujian, Gansu, Hubei, Sichuan.
43. *T. reticulatus* (Chang & He, 2001), Palearctic and Oriental Regions: China: Beijing, Gansu, Inner Mongolia, Jilin, Ningxia, Qinghai, Shaanxi, Yunnan.
44. *T. ruginosus* Zhou et al., 2010, Oriental Region: China: Guizhou, Hunan, Zhejiang.
45. *T. shannxiensis* Zhou et al., 2010, Palearctic Region: China: Shaanxi.
46. *T. shennongi* sp. nov., Oriental Region: China: Hubei.
47. *T. shuangjiang* Qian & Xu, 2024, Oriental Region: China: Yunnan.
48. *T. spinosior* (Forel, 1901), Palearctic and Oriental Regions: China: Anhui, Beijing, Guangdong, Guangxi, Hebei, Henan, Hubei, Hunan, Ningxia, Shaanxi, Shandong, Shanxi, Sichuan, Yunnan, Zhejiang; Japan, South Korea.
49. *T. striatus* Zhou et al., 2010, Palearctic and Oriental Regions: China: Henan, Hubei, Ningxia, Sichuan, Yunnan.
50. *T. susamyri* (Dlussky, 1965), Palearctic Region: China: Xizang; Kyrgyzstan.
51. *T. taivanensis* (Wheeler, 1929), Oriental Region: China: Fujian, Guangdong, Guangxi, Hainan, Hunan, Taiwan, Yunnan.
52. *T. tianpeng* Terayama, 2009, Oriental Region: China: Taiwan.
53. *T. volgensis* (Ruzsky, 1905), Palearctic Region: China: Qinghai; Russia.
54. *T. wangweii* sp. nov., Oriental Region: China: Hubei.
55. *T. wui* (Wheeler, 1929), Palearctic and Oriental Regions: China: Beijing, Hebei, Hunan, Sichuan, Yunnan.
56. *T. xanthos* Radchenko, 2004, Palearctic Region: China: Jiangsu; North Korea.
57. *T. xia* Qian & Xu, 2024, Palearctic and Oriental Regions: China: Xizang, Yunnan.
58. *T. xiaohan* Qian & Xu, 2024, Palearctic Region: China: Xizang.
59. *T. xiaoman* Qian & Xu, 2024, Palearctic Region: China: Shaanxi.
60. *T. xiaoshu* Qian & Xu, 2024, Oriental Region: China: Yunnan.
61. *T. xiaoxue* Qian & Xu, 2024, Oriental Region: China: Yunnan.
62. *T. xiazhi* Qian & Xu, 2024, Oriental Region: China: Yunnan.
63. *T. yanwan* Terayama, 2009, Oriental Region: China: Taiwan.
64. *T. yushui* Qian & Xu, 2024, Oriental Region: China: Sichuan, Yunnan.
65. *T. zhaojunae* sp. nov., Oriental Region: China: Hubei.
66. *T. zhejiangensis* Zhou et al., 2010, Oriental Region: China: Guangxi, Henan, Zhejiang.

### Groups of Chinese *Temnothorax* species

To facilitate identification of the 66 known Chinese *Temnothorax* species, we divided them into 20 species groups. Diagnostic characters for each group with the related species are provided below.

#### 1 koreanus group

**Diagnosis**. Species of this group are characterized by the following combination of characters: antennae 11-segmented; humeral corners of pronotum bluntly angled in dorsal view; metanotal groove absent; dorsum of mesonotum and propodeum weakly convex; posterodorsal corner of propodeum spined in lateral view, with spines approximately as long as declivity; petiole with a distinct anterior peduncle, shorter than petiolar node; petiolar node trapezoidal with a broadly rounded dorsum in lateral view; head, mesosoma, petiole and postpetiole coarsely reticulate except head dorsum mainly rugose; body dorsum with sparse apically blunt hairs, scapes and tibiae without standing hairs. Included one species: *T. koreanus* (Teranishi, 1940).

#### 2 angulohumerus group

**Diagnosis**. Species of this group are characterized by the following combination of characters: antennae 12-segmented; humeral corners of pronotum bluntly to right-angled in dorsal view; metanotal groove absent; dorsum of mesonotum and propodeum nearly straight; posterodorsal corner of propodeum spined in lateral view, with spines shorter than declivity; petiole with a distinct anterior peduncle, approx. as long as petiolar node; petiolar node trapezoidal with a broadly rounded dorsum in lateral view; head dorsum loosely rugose with central area nearly smooth; mesosoma and petiole mainly reticulate except pronotal sides rugose; body dorsum with sparse or very sparse apically blunt hairs, scapes and tibiae with or without decumbent hairs. Included three species: *T. angulohumerus* Zhou et al., 2010, *T. bailu* Qian & Xu, 2024 and *T. orchidus* Zhou et al., 2010.

#### 3 hengshanensis group

**Diagnosis**. Species of this group are characterized by the following combination of characters: antennae 12-segmented; humeral corners of pronotum narrowly to broadly rounded in dorsal view; metanotal groove absent; dorsum of mesonotum and propodeum weakly convex; posterodorsal corner of propodeum rounded in lateral view, without spines; petiole with a distinct anterior peduncle, shorter than petiolar node; petiolar node nearly triangular or trapezoidal, with a bluntly angled or broadly rounded dorsum in lateral view; head, mesosoma and petiole reticulate and punctate; body dorsum with sparse or very sparse apically blunt hairs, scapes and tibiae without standing hairs. Included two species: *T. hengshanensis* (Huang et al., 2004) and *T. leimu* Terayama, 2009.

#### 4 maoerensis group

**Diagnosis**. Species of this group are characterized by the following combination of characters: antennae 12-segmented; humeral corners of pronotum broadly rounded in dorsal view; metanotal groove present; dorsum of mesonotum and propodeum nearly straight; posterodorsal corner of propodeum bluntly angled to triangularly toothed in lateral view, the teeth shorter than their basal width; petiole with a distinct anterior peduncle, shorter than petiolar node; petiolar node approx. conical or trapezoidal, with a narrowly rounded dorsum in lateral view; head, mesosoma, petiole and postpetiole densely or loosely reticulate; body dorsum with sparse apically blunt hairs, scapes and tibiae with decumbent to subdecumbent standing hairs. Included four species: *T. chushu* Qian & Xu, 2024, *T. dashu* Qian & Xu, 2024, *T. maoerensis* Zhou et al., 2010 and *T. yushui* Qian & Xu, 2024.

#### 5 congruus group

**Diagnosis**. Species of this group are characterized by the following combination of characters: antennae 12-segmented; humeral corners of pronotum broadly rounded in dorsal view; metanotal groove absent; dorsum of mesonotum and propodeum weakly convex or nearly straight; posterodorsal corner of propodeum bluntly angled to triangularly toothed in lateral view, the teeth shorter than their basal width; petiole with a distinct anterior peduncle, shorter than petiolar node; petiolar node approx. triangular or semicircular, with a bluntly angled or narrowly rounded dorsum in lateral view; head, mesosoma and petiole densely reticulate except head dorsum mainly rugose; body dorsum with sparse apically blunt or acute hairs, scapes and tibiae with or without decumbent to subdecumbent standing hairs. Included three species: *T. congruus* (Smith, 1874), *T. jingzhe* Qian & Xu, 2024 and *T. mangzhong* Qian & Xu, 2024.

#### 6 chunfen group

**Diagnosis**. Species of this group are characterized by the following combination of characters: antennae 12-segmented; humeral corners of pronotum broadly rounded in dorsal view; metanotal groove absent; dorsum of mesonotum and propodeum weakly convex or nearly straight; posterodorsal corner of propodeum triangularly toothed in lateral view, the teeth shorter than their basal width; petiole with a distinct anterior peduncle, shorter than petiolar node; petiolar node approx. conical, with a narrowly rounded dorsum in lateral view; head wholly or mostly smooth; mesosoma, petiole and postpetiole densely reticulate; body dorsum with sparse apically blunt hairs, scapes and tibiae without decumbent standing hairs. Included two species: *T. chunfen* Qian & Xu, 2024 and *T. guyu* Qian & Xu, 2024.

#### 7 desioi group

**Diagnosis**. Species of this group are characterized by the following combination of characters: antennae 12-segmented; humeral corners of pronotum broadly rounded in dorsal view; metanotal groove absent; dorsum of mesonotum and propodeum nearly straight; posterodorsal corner of propodeum bluntly angled in lateral view; petiole without a distinct anterior peduncle; petiolar node approx. triangular, with a right-angled dorsum in lateral view; head almost smooth with a few rugae; mesosoma, petiole and postpetiole densely reticulate; body dorsum with sparse apically blunt hairs, scapes and tibiae without decumbent standing hairs. Included one species: *T. desioi* (Menozzi, 1939).

#### 8 liqiu group

**Diagnosis**. Species of this group are characterized by the following combination of characters: antennae 12-segmented; humeral corners of pronotum broadly rounded in dorsal view; metanotal groove present; dorsum of mesonotum and propodeum weakly convex or nearly straight; posterodorsal corner of propodeum shortly toothed or spined in lateral view, the teeth or spines longer than their basal width but shorter than declivity; petiole with a distinct anterior peduncle, shorter than or as long as petiolar node; petiolar node approx. conical or trapezoidal, with a narrowly rounded dorsum in lateral view; head, mesosoma, petiole and postpetiole densely reticulate or rugose; body dorsum with sparse apically acute hairs, scapes and tibiae with or without decumbent standing hairs. Included three species: *T. liqiu* Qian & Xu, 2024, *T. shennongi* sp. nov. and *T. xiaoshu* Qian & Xu, 2024.

#### 9 wui group

**Diagnosis**. Species of this group are characterized by the following combination of characters: antennae 12-segmented; humeral corners of pronotum broadly rounded in dorsal view; metanotal groove absent; dorsum of mesonotum and propodeum weakly convex or nearly straight; posterodorsal corner of propodeum shortly toothed or spined in lateral view, the teeth or spines longer than their basal width but shorter than declivity; petiole with a distinct anterior peduncle, shorter than petiolar node; petiolar node approx. conical or triangular, with a narrowly rounded or right-angled dorsum in lateral view; head wholly or mostly smooth on the central area; mesosoma, petiole and postpetiole densely reticulate or rugose; body dorsum with sparse apically blunt hairs, scapes and tibiae without subdecumbent standing hairs. Included three species: *T. qingming* Qian & Xu, 2024, *T. susamyri* (Dlussky, 1965) and *T. wui* (Wheeler, 1929).

#### 10 mongolicus group

**Diagnosis**. Species of this group are characterized by the following combination of characters: antennae 12-segmented; humeral corners of pronotum broadly rounded in dorsal view; metanotal groove absent; dorsum of mesonotum and propodeum weakly convex; posterodorsal corner of propodeum shortly spined in lateral view, the spines longer than their basal width but shorter than declivity; petiole without anterior peduncle; petiolar node approx. triangular, with a bluntly angled dorsum in lateral view; head, mesosoma, petiole and postpetiole densely reticulate; body dorsum with sparse apically blunt hairs, scapes and tibiae without subdecumbent standing hairs. Included two species: *T. brevispinus* (Chang & He, 2001) and *T. mongolicus* (Pisarski, 1969).

#### 11 leyeensis group

**Diagnosis**. Species of this group are characterized by the following combination of characters: antennae 12-segmented; humeral corners of pronotum broadly rounded in dorsal view; metanotal groove absent; dorsum of mesonotum and propodeum weakly convex or nearly straight; posterodorsal corner of propodeum shortly spined in lateral view, the spines longer than their basal width but shorter than declivity; petiole with a distinct anterior peduncle, shorter than petiolar node; petiolar node approx. trapezoidal, with a narrowly rounded dorsum in lateral view; head sides, mesosoma, petiole and postpetiole densely reticulate, head dorsum rugose or reticulate; body dorsum with sparse or very sparse apically blunt or acute hairs, scapes and tibiae with or without subdecumbent standing hairs. Included five species: *T. chun* Qian & Xu, 2024, *T. dongzhi* Qian & Xu, 2024, *T. leyeensis* Zhou et al., 2010, *T. xia* Qian & Xu, 2024 and *T. xiaoxue* Qian & Xu, 2024.

#### 12 spinosior group

**Diagnosis**. Species of this group are characterized by the following combination of characters: antennae 12-segmented; humeral corners of pronotum broadly rounded in dorsal view; metanotal groove absent; dorsum of mesonotum and propodeum weakly convex; posterodorsal corner of propodeum shortly spined in lateral view, the spines longer than their basal width but shorter than declivity; petiole with a distinct anterior peduncle, shorter than petiolar node; petiolar node symmetrical, approx. conical or triangular, with a bluntly or right-angled dorsum in lateral view; head sides, mesosoma, petiole and postpetiole densely reticulate, head dorsum rugose or reticulate; body dorsum with sparse or very sparse apically blunt hairs, scapes and tibiae without subdecumbent standing hairs. Included seven species: *T. dahan* Qian & Xu, 2024, *T. dong* Qian & Xu, 2024, *T. huatuo* Terayama, 2009, *T. pisarskii* Radchenko, 2004, *T. qiu* Qian & Xu, 2024, *T. shuangjiang* Qian & Xu, 2024 and *T. spinosior* (Forel, 1901).

#### 13 striatus group

**Diagnosis**. Species of this group are characterized by the following combination of characters: antennae 12-segmented; humeral corners of pronotum broadly rounded in dorsal view; metanotal groove absent; dorsum of mesonotum and propodeum weakly convex or nearly straight; posterodorsal corner of propodeum shortly spined in lateral view, the spines longer than their basal width but shorter than declivity; petiole with a distinct anterior peduncle, shorter than or as long as petiolar node; petiolar node asymmetric with more convex posterior margin, approx. triangular, with a bluntly or right-angled dorsum in lateral view; head sides, mesosoma, petiole and postpetiole densely reticulate, head dorsum rugose; body dorsum with sparse apically blunt or acute hairs, scapes and tibiae with or without decumbent standing hairs. Included seven species: *T. lixia* Qian & Xu, 2024, *T. qiufen* Qian & Xu, 2024, *T. shannxiensis* Zhou et al., 2010, *T. striatus* Zhou et al., 2010, *T. xiaohan* Qian & Xu, 2024, *T. xiaoman* Qian & Xu, 2024 and *T. xiazhi* Qian & Xu, 2024.

#### 14 confucii group

**Diagnosis**. Species of this group are characterized by the following combination of characters: antennae 12-segmented; humeral corners of pronotum broadly rounded in dorsal view; metanotal groove present; dorsum of mesonotum and propodeum concave at metanotal groove; posterodorsal corner of propodeum with long spines in lateral view, the spines approx. as long as or longer than declivity; petiole with a distinct anterior peduncle, longer than petiolar node; petiolar node approx. triangular, with a right-or bluntly angled dorsum in lateral view; head and mesosoma loosely rugose or densely reticulate, head sides reticulate, petiole and postpetiole densely punctate; body dorsum with sparse to abundant apically blunt or acute hairs, scapes and tibiae without subdecumbent standing hairs. Included three species: *T. argentipes* (Wheeler, 1928), *T. confucii* (Forel, 1912) and *T. haveni* Lee et al., 2023.

#### 15 kuixing group

**Diagnosis**. Species of this group are characterized by the following combination of characters: antennae 12-segmented; humeral corners of pronotum broadly rounded in dorsal view; metanotal groove present; dorsum of mesonotum and propodeum concave at metanotal groove; posterodorsal corner of propodeum with long spines in lateral view, the spines approx. as long as or longer than declivity; petiole with a distinct anterior peduncle, as long as or shorter than petiolar node; petiolar node approx. conical or triangular, with a narrowly rounded or bluntly angled dorsum in lateral view; head reticulate or rugose, mesosoma reticulate, petiole and postpetiole densely punctate; body dorsum with sparse apically blunt hairs, scapes and tibiae with or without decumbent standing hairs. Included three species: *T. kuixing* Terayama, 2009, *T. leigong* Terayama, 2009 and *T. zhaojunae* sp. nov.

#### 16 lichun group

**Diagnosis**. Species of this group are characterized by the following combination of characters: antennae 12-segmented; humeral corners of pronotum narrowly rounded in dorsal view; metanotal groove present; dorsum of mesonotum and propodeum concave at metanotal groove; posterodorsal corner of propodeum with long spines in lateral view, the spines longer than declivity; petiole with a distinct anterior peduncle, shorter than petiolar node; petiolar node approx. trapezoidal, with a narrowly rounded dorsum in lateral view; head, mesosoma, petiole and postpetiole densely or loosely reticulate; body dorsum with abundant apically blunt hairs, scapes and tibiae without subdecumbent standing hairs. Included two species: *T. lichun* Qian & Xu, 2024 and *T. wangweii* sp. nov.

#### 17 taivanensis group

**Diagnosis**. Species of this group are characterized by the following combination of characters: antennae 12-segmented; humeral corners of pronotum narrowly rounded in dorsal view; metanotal groove absent; dorsum of mesonotum and propodeum weakly convex; posterodorsal corner of propodeum with long spines in lateral view, the spines approx. as long as declivity; petiole with a distinct anterior peduncle, longer than petiolar node; petiolar node approx. conical, with a narrowly rounded dorsum in lateral view; head and mesosoma rogose, petiole and postpetiole densely punctate; body dorsum with abundant apically acute hairs, scapes and tibiae without subdecumbent standing hairs. Included one species: *T. taivanensis* (Wheeler, 1929).

#### 18 reduncus group

**Diagnosis**. Species of this group are characterized by the following combination of characters: antennae 12-segmented; humeral corners of pronotum narrowly to broadly rounded in dorsal view; metanotal groove absent; dorsum of mesonotum and propodeum nearly straight; posterodorsal corner of propodeum with long spines in lateral view, the spines approx. as long as declivity; petiole with a distinct anterior peduncle, shorter than or as long as petiolar node; petiolar node approx. conical or triangular, with a narrowly rounded or bluntly angled dorsum in lateral view; head reticulate or rugose, mesosoma reticulate, petiole and postpetiole densely punctate; body dorsum with sparse or abundant apically blunt or acute hairs, scapes and tibiae without subdecumbent standing hairs. Included five species: *T. barrettoi* Hamer & Guénard, 2023, *T. lidong* Qian & Xu, 2024, *T. reduncus* (Wang & Wu, 1988), *T. tianpeng* Terayama, 2009 and *T. yanwan* Terayama, 2009.

#### 19 volgensis group

**Diagnosis**. Species of this group are characterized by the following combination of characters: antennae 12-segmented; humeral corners of pronotum narrowly to broadly rounded in dorsal view; metanotal groove absent; dorsum of mesonotum and propodeum weakly convex; posterodorsal corner of propodeum with long spines in lateral view, the spines approx. as long as declivity; petiole with a distinct anterior peduncle, shorter than petiolar node; petiolar node approx. conical or triangular, with a narrowly rounded or bluntly angled dorsum in lateral view; head sides, mesosoma, petiole and postpetiole reticulate, head dorsum reticulate or rugose; body dorsum with sparse or very sparse apically blunt or acute hairs, scapes and tibiae with or without decumbent standing hairs. Included six species: *T. daxue* Qian & Xu, 2024, *T. reticulatus* (Chang & He, 2001), *T. ruginosus* Zhou et al., 2010, *T. volgensis* (Ruzsky, 1905), *T. xanthos* Radchenko, 2004 and *T. zhejiangensis* Zhou et al., 2010.

#### 20 eburneipes group

**Diagnosis**. Species of this group are characterized by the following combination of characters: antennae 12-segmented; humeral corners of pronotum narrowly rounded in dorsal view; metanotal groove absent; dorsum of mesonotum and propodeum weakly convex; posterodorsal corner of propodeum with long spines in lateral view, the spines longer than declivity; petiole with a distinct anterior peduncle, shorter than petiolar node; petiolar node approx. triangular or trapezoidal, with a narrowly rounded or bluntly angled dorsum in lateral view; head sides and mesosoma reticulate, head dorsum rugose or reticulate, petiole and postpetiole punctate; body dorsum with sparse or abundant apically blunt or acute hairs, scapes and tibiae without subdecumbent standing hairs. Included three species: *T. eburneipes* (Wheeler, 1927), *T. hanlu* Qian & Xu, 2024 and *T. hubeiensis* sp. nov.

### Key to the known Chinese *Temnothorax* species (worker caste)

This key to the 66 known Chinese species of *Temnothorax* is revised from that of Qian and Xu (2024) based on the species grouping to incorporate the four new species described in the present study. In the original key of Qian and Xu (2024), *Temnothorax taivanensis* (Wheeler, 1929) was incorrectly placed due to the misinterpretation of the absence of the metanotal groove. In the revised key presented here, this species is positioned correctly based on that diagnostic character.

**Table:** 

1	Antennae 11-segmented [Palearctic Region: China: Hubei; North Korea, Japan, South Korea (type locality)] (see Qian and Xu 2024: fig. 48)	***T. koreanus* (Teranishi, 1940)**
–	Antennae 12-segmented	**2**
2	Humeral corners of pronotum protruding, right-to bluntly angled in dorsal view	**3**
–	Humeral corners of pronotum narrowly to broadly rounded in dorsal view	**5**
3	Humeral corners of pronotum bluntly angled in dorsal view. Dorsum of petiolar node sloping down posteriorly. Body color blackish brown, head and gaster black [Palearctic and Oriental Regions: China: Sichuan, Xizang, Yunnan] (see Qian and Xu 2024: fig. 3)	***T. bailu* Qian & Xu, 2024**
–	Humeral corners of pronotum right-angled in dorsal view. Dorsum of petiolar node almost horizontal. Body color yellowish brown, head and gaster blackish brown	**4**
4	In full-face view antennal scapes surpassing posterior head margin. Pronotum strongly convex in lateral view. Propodeal spines ~ 2× as long as their basal width in lateral view, tips slightly curved posterodorsally [Palearctic and Oriental Regions: China: Guangxi, Guizhou, Hunan, Xizang, Yunnan] (see Qian and Xu 2024: fig. 4)	***T. angulohumerus* Zhou et al., 2010**
–	In full-face view antennal scapes fail to reach posterior head margin. Pronotum weakly convex in lateral view. Propodeal spines ~ 1.2× as long as their basal width in lateral view, tips strongly curved posteriorly [Oriental Region: China: Guangxi, Sichuan, and Yunnan] (see Qian and Xu 2024: fig. 51)	***T. orchidus* Zhou et al., 2010**
5	Posterodorsal corner of propodeum rounded in lateral view (see Qian and Xu 2024: figs 49, 50)	**6**
–	Posterodorsal corner of propodeum bluntly to right-angled (see Qian and Xu 2024: figs 19B, 20B), toothed (see Qian and Xu 2024: figs 27, 38) or spined (see Qian and Xu 2024: figs 23, 24) in lateral view	**7**
6	Posterior head margin moderately convex. Posterodorsal corner of propodeum narrowly rounded in lateral view. Petiolar node approx. triangular and without obvious dorsal margin [Oriental Region: China: Hunan, Yunnan] (see Qian and Xu 2024: fig. 49)	***T. hengshanensis* (Huang et al., 2004)**
–	Posterior head margin slightly convex. Posterodorsal corner of propodeum broadly rounded in lateral view. Petiolar node approx. trapezoidal and with obvious dorsal margin [Oriental Region: China: Taiwan (type locality)] (see Qian and Xu 2024: fig. 50)	***T. leimu* Terayama, 2009**
7	Posterodorsal corner of propodeum bluntly angled or triangularly toothed, if toothed than the teeth not longer than their basal width in lateral view	**8**
–	Posterodorsal corner of propodeum as short- to long-spined, the spines longer than their basal width in lateral view	**17**
8	Metanotal groove obviously impressed	**9**
–	Metanotal groove absent	**12**
9	Posterodorsal corner of propodeum bluntly angled [Oriental Region: China: Sichuan, Yunnan] (see Qian and Xu 2024: fig. 43)	***T. yushui* Qian & Xu, 2024**
–	Posterodorsal corner of propodeum triangularly toothed	**10**
10	Anterior margin of petiolar node nearly straight [Palearctic and Oriental Region: China: Xizang, Yunnan] (see Qian and Xu 2024: fig. 12)	***T. dashu* Qian & Xu, 2024**
–	Anterior margin of petiolar node obviously concave	**11**
11	Head dorsum weakly reticulate and relatively smooth. Body color blackish brown [Oriental Region: China: Guangxi (type locality)] (see Qian and Xu 2024: fig. 9)	***T. maoerensis* Zhou et al., 2010**
–	Head dorsum strongly reticulate and dull. Body color brownish yellow [Oriental Region: China: Sichuan, Yunnan] (see Qian and Xu 2024: fig. 8)	***T. chushu* Qian & Xu, 2024**
12	Petiole without anterior peduncle [Palearctic and Oriental Regions: China: Xizang; India (type locality), Pakistan] (see Qian and Xu 2024: fig. 20)	***T. desioi* (Menozzi, 1939)**
–	Petiole with distinct anterior peduncle	**13**
13	Head dorsum wholly smooth or at least smooth on the longitudinal central strip	**14**
–	Head dorsum rugose or reticulate	**15**
14	Head dorsum wholly smooth. Dorsum of mesonotum and propodeum nearly straight in lateral view [Oriental Region: China: Yunnan] (see Qian and Xu 2024: fig. 6)	***T. chunfen* Qian & Xu, 2024**
–	Head dorsum smooth on the longitudinal central strip, rugose and reticulate laterally. Dorsum of mesonotum and propodeum weakly convex in lateral view [Oriental Region: China: Sichuan and Yunnan] (see Qian and Xu 2024: fig. 19)	***T. guyu* Qian & Xu, 2024**
15	Posterodorsal corner of propodeum right--angled. Petiolar node nearly semicircular in lateral view [Oriental Region: China: Yunnan] (see Qian and Xu 2024: fig. 29)	***T. mangzhong* Qian & Xu, 2024**
–	Posterodorsal corner of propodeum triangularly toothed. Petiolar node approx. triangular or conical in lateral view [Oriental Region: China: Yunnan] (see Qian and Xu 2024: fig. 29)	**16**
16	Petiolar node approx. triangular and weakly inclined anteriorly, with summit bluntly angled. Head dorsum coarsely longitudinally rugose. Body color reddish brown [Palearctic and Oriental Regions: China: Beijing, Guangxi, Yunnan; North Korea, Japan (type locality), South Korea, Russia] (see Qian and Xu 2024: fig. 7)	***T. congruus* (Smith, 1874)**
–	Petiolar node approx. conical and erect, with summit narrowly rounded. Head dorsum finely longitudinally rugose. Body color brownish black [Oriental Region: China: Yunnan] (see Qian and Xu 2024: fig. 22)	***T. jingzhe* Qian & Xu, 2024**
17	Posterodorsal corner of propodeum shortly spined, the spines shorter than declivity	**18**
–	Posterodorsal corner of propodeum long-spined, the spines as long as or longer than declivity	**44**
18	Metanotal groove obviously impressed	**19**
–	Metanotal groove absent	**21**
19	Petiolar node approx. conical, with narrowly rounded summit. Head dorsum coarse reticulate [Oriental Region: China: Yunnan] (fig. 3)	***T. xiaoshu* Qian & Xu, 2024**
–	Petiolar node approx. trapezoidal, with obvious dorsal margin. Head dorsum finely reticulate or longitudinally rugose	**20**
20	Propodeal spines very short, ~ 1.5× as long as their basal width. Head dorsum longitudinally rugose anteriorly and reticulate posteriorly, mesosoma densely reticulate. Body color yellowish brown, head brownish black [Oriental Region: China: Sichuan, Yunnan] (fig. 2)	***T. liqiu* Qian & Xu, 2024**
–	Propodeal spines relatively longer, ~ 3× as long as their basal width. Head dorsum loosely longitudinally rugose, mesosoma mainly longitudinally rugose. Body black, mesosoma, petiole and postpetiole brownish black [Oriental Region: China: Hubei] (fig. 1)	***T. shennongi* sp. nov**.
21	Head dorsum wholly smooth or at least smooth on the longitudinal central strip	**22**
–	Head dorsum wholly sculptured	**24**
22	Head dorsum wholly smooth. Petiolar node approx. conical with narrowly rounded summit [Palearctic Region: China: Xizang] (see Qian and Xu 2024: fig. 30)	***T. qingming* Qian & Xu, 2024**
–	Head dorsum smooth on the longitudinal central strip, rugose or reticulate laterally. Petiolar node approx. triangular with right-angled summit	**23**
23	Dorsum of mesonotum and propodeum nearly straight in lateral view [Palearctic and Oriental Regions: China: Beijing, Hebei, Hunan, Sichuan, Yunnan] (see Qian and Xu 2024: fig. 31)	***T. wui* (Wheeler, 1929)**
–	Dorsum of mesonotum and propodeum weakly convex in lateral view [Palearctic Region: China: Xizang; Kyrgyzstan (type locality)] (see Qian and Xu 2024: fig. 65)	***T. susamyri* (Dlussky, 1965)**
24	Petiole without anterior peduncle	**25**
–	Petiole with distinct anterior peduncle	**26**
25	Head dorsum longitudinally rugose on the central area. Propodeal spines thin in lateral view. Body color yellow, head and gaster blackish brown [Palearctic Region: China: Hebei, Inner Mongolia; North Korea, Mongolia (type locality), Russia] (see Qian and Xu 2024: fig. 1)	***T. mongolicus* (Pisarski, 1969)**
–	Head dorsum finely reticulate on the central area. Propodeal spines thick in lateral view. Body color reddish brown, head and gaster brownish black [Palearctic Region: China: Ningxia, Qinghai] (see Qian and Xu 2024: fig. 66)	***T. brevispinus* (Chang & He, 2001)**
26	Petiolar node approx. trapezoidal, with obvious dorsal margin	**27**
–	Petiolar node approx. triangular or conical, without obvious dorsal margin	**31**
27	Dorsum of mesonotum and propodeum weakly convex	**28**
–	Dorsum of mesonotum and propodeum nearly straight	**29**
28	Head dorsum densely reticulate. Propodeal spines posterodorsally pointed, formed a blunt angle with propodeal dorsum. Body color black, mesosoma, petiole and postpetiole brownish yellow [Oriental Region: China: Guangxi (type locality)] (see Qian and Xu 2024: fig. 18)	***T. leyeensis* Zhou et al., 2010**
–	Head dorsum longitudinally rugose. Propodeal spines suberect, formed a right angle with propodeal dorsum. Body color black, mesosoma, petiole, and postpetiole blackish brown [Oriental Region: China: Sichuan] (see Qian and Xu 2024: fig. 17)	***T. dongzhi* Qian & Xu, 2024**
29	Scapes with subdecumbent hairs. Sides of pronotum reticulate [Oriental Region: China: Yunnan] (see Qian and Xu 2024: fig. 39)	***T. xiaoxue* Qian & Xu, 2024**
–	Scapes without subdecumbent hairs. Sides of pronotum longitudinally rugose	**30**
30	Head dorsum loosely longitudinally rugose. Petiolar node smooth. Body color black [Oriental Region: China: Yunnan] (see Qian and Xu 2024: fig. 5)	***T. chun* Qian & Xu, 2024**
–	Head dorsum densely longitudinally rugose. Petiolar node densely reticulate. Body color blackish brown [Palearctic and Oriental Regions: China: Xizang, Yunnan] (see Qian and Xu 2024: fig. 35)	***T. xia* Qian & Xu, 2024**
31	Petiolar node nearly symmetrical in lateral view, posterior margin approx. as convex as anterior margin	**32**
–	Petiolar node asymmetrical in lateral view, posterior margin more convex than anterior margin	**38**
32	Anterior margin of petiolar node nearly straight	**33**
–	Anterior margin of petiolar node obviously concave	**35**
33	Petiolar node approx. conical, with narrowly rounded summit. Body color blackish brown [Palearctic and Oriental Regions: China: Anhui, Beijing, Guangdong, Guangxi, Hebei, Henan, Hubei, Hunan, Ningxia, Shaanxi, Shandong, Shanxi, Sichuan, Yunnan, Zhejiang; Japan (type locality), South Korea] (see Qian and Xu 2024: fig. 11)	***T. spinosior* (Forel, 1901)**
–	Petiolar node approx. triangular, with right-angled summit. Body color blackish or yellow	**34**
34	Head dorsum densely longitudinally rugose. Body color yellow [Palearctic Region: China: Hebei, Heilongjiang, Liaoning, Shaanxi; North Korea] (see Qian and Xu 2024: fig. 67)	***T. pisarskii* Radchenko, 2004**
–	Head dorsum loosely longitudinally rugose. Body color black [Oriental Region: China: Yunnan] (see Qian and Xu 2024: fig. 10)	***T. dahan* Qian & Xu, 2024**
35	Petiolar node approx. conical, with narrowly rounded summit	**36**
–	Petiolar node approx. triangular, with right- angled summit	**37**
36	Head dorsum parallelly rugose. Mesosoma finely reticulate [Oriental Region: China: Sichuan, Yunnan] (see Qian and Xu 2024: fig. 32)	***T. qiu* Qian & Xu, 2024**
–	Head dorsum irregularly rugose. Mesosoma coarsely reticulate [Oriental Region: China: Yunnan] (see Qian and Xu 2024: fig. 34)	***T. shuangjiang* Qian & Xu, 2024**
37	Head dorsum longitudinally rugose on the central area. Body color brownish black [Oriental Region: China: Taiwan (type locality)] (See Qian and Xu 2024: fig. 16)	***T. huatuo* Terayama, 2009**
–	Head dorsum densely reticulate on the central area. Body color black [Oriental Region: China: Yunnan] (see Qian and Xu 2024: fig. 15)	***T. dong* Qian & Xu, 2024**
38	Anterior margin of petiolar node near straight in lateral view	**39**
–	Anterior margin of petiolar node obviously concave in lateral view	**40**
39	Head dorsum mainly longitudinally rugose, the rugae almost reaching to posterior head margin. Sides of pronotum coarsely reticulate [Palearctic and Oriental Regions: China: Henan, Hubei, Ningxia, Sichuan, Yunnan] (see Qian and Xu 2024: fig. 40)	***T. striatus* Zhou et al., 2010**
–	Head dorsum longitudinally rugose in the anterior half and reticulate in the posterior half. Sides of pronotum longitudinally rugose [Palearctic Region: China: Shaanxi] (see Qian and Xu 2024: fig. 64)	***T. shannxiensis* Zhou et al., 2010**
40	Pronotal sides finely reticulate	41
–	Pronotal sides coarsely reticulate	42
41	Head dorsum loosely longitudinally rugose [Palearctic and Oriental Regions: China: Sichuan, Qinghai] (see Qian and Xu 2024: fig. 33)	***T. qiufen* Qian & Xu, 2024**
–	Head dorsum densely longitudinally rugose [Oriental Region: China: Yunnan] (see Qian and Xu 2024: fig. 41)	***T. xiazhi* Qian & Xu, 2024**
42	Dorsum of mesonotum and propodeum weakly convex [Oriental Region China: Sichuan] (see Qian and Xu 2024: fig. 28)	***T. lixia* Qian & Xu, 2024**
–	Dorsum of mesonotum and propodeum nearly straight	**43**
43	Head dorsum reticulate. Propodeal spines nearly suberect. Petiolar node with right-angled summit [Palearctic Region: China: Shaanxi] (see Qian and Xu 2024: fig. 37)	***T. xiaoman* Qian & Xu, 2024**
–	Head dorsum longitudinally rugose. Propodeal spines posterodorsally pointed. Petiolar node with narrowly rounded summit [Palearctic Region: China: Xizang] (see Qian and Xu 2024: fig. 36)	***T. xiaohan* Qian & Xu, 2024**
44	Metanotal groove obviously impressed	**45**
–	Metanotal groove absent	**52**
45	Anterior peduncle longer than petiolar node	**46**
–	Anterior peduncle as long as or shorter than petiolar node	**48**
46	Petiolar node asymmetrical, posterior margin much convex than anterior margin [Oriental Region: China: Taiwan (type locality)] (see Qian and Xu 2024: fig. 55)	***T. confucii* (Forel, 1912)**
–	Petiolar node symmetrical, posterior margin as convex as anterior margin	**47**
47	Propodeal spines slender and slightly down-curved, longer than declivity [Palearctic and Oriental Regions: China: Beijing, Fujian, Guangxi, Hebei, Henan, Jiangxi, Liaoning, Ningxia, Shaanxi, Sichuan, Yunnan] (see Qian and Xu 2024: fig. 42)	***T. argentipes* (Wheeler, 1928)**
–	Propodeal spines stout and strongly down-curved, as long as declivity [Oriental Region: China: Hong Kong (type locality)] (see Qian and Xu 2024: fig. 52)	***T. haveni* Lee et al., 2023**
48	Petiolar node approx. trapezoidal, with distinct dorsal margin	**49**
–	Petiolar node approx. conical or triangular, with narrowly rounded or right-angled summit	**50**
49	Head dorsum coarsely reticulate. Anterior margin of clypeus concave in the center. Promesonotum weakly convex. Anterior peduncle ~ 2/5 length of petiolar node. Head and gaster black, mesosoma, petiole, and postpetiole reddish brown [Oriental Region: China: Yunnan] (fig. 5)	***T. lichun* Qian & Xu, 2024**
–	Head dorsum loosely longitudinally rugose. Anterior margin of clypeus convex in the center. Promesonotum strongly convex. Anterior peduncle ~ 1/3 length of petiolar node. Body color black [Oriental Region: China: Hubei] (fig. 4)	***T. wangweii* sp. nov**.
50	Petiolar node approx. triangular, anterior margin nearly straight, with right-angled summit. Head dorsum longitudinally rugose [Oriental Region: China: Hubei] (fig. 10)	***T. zhaojunae* sp. nov**.
–	Petiolar node approx. conical, anterior margin obviously concave, with narrowly rounded summit. Head dorsum densely reticulate	**51**
51	Anterior peduncle approx. as long as petiolar node in lateral view. Head dorsum densely punctate with loose longitudinal rugae. Body color brownish yellow, head blackish brown, gaster black [Oriental Region: China: Taiwan (type locality)] (see Qian and Xu 2024: fig. 53)	***T. leigong* Terayama, 2009**
–	Anterior peduncle shorter than petiolar node in lateral view. Head dorsum finely reticulate. Body color blackish brown, gaster black [Oriental Region: China: Taiwan (type locality)] (fig. 6)	***T. kuixing* Terayama, 2009**
52	Anterior peduncle obviously longer than petiolar node [Oriental Region: China: Fujian, Guangdong, Guangxi, Hainan, Hunan, Taiwan, Yunnan] (see Qian and Xu 2024: fig. 24)	***T. taivanensis* (Wheeler, 1929)**
–	Anterior peduncle as long as or shorter than petiolar node	**53**
53	Dorsum of mesonotum and propodeum nearly straight in lateral view	**54**
–	Dorsum of mesonotum and propodeum weakly to moderately convex in lateral view	**58**
54	Anterior margin of petiolar node obviously concave	**55**
–	Anterior margin of petiolar node nearly straight	**56**
55	Head dorsum loosely reticulate. Promesonotum weakly convex [Palearctic and Oriental Regions: China: Fujian, Gansu, Hubei, Sichuan] (see Qian and Xu 2024: fig. 62)	***T. reduncus* (Wang & Wu, 1988)**
–	Head dorsum mainly longitudinally rugose with interface densely punctate. Promesonotum moderately convex [Oriental Region: China: Taiwan (type locality)] (see Qian and Xu 2024: fig. 63)	***T. tianpeng* Terayama, 2009**
56	Propodeal spines obviously longer than declivity. Head dorsum reticulate. Body color yellow [Oriental Region: China: Hong Kong (type locality)] (see Qian and Xu 2024: fig. 61)	***T. barrettoi* Hamer & Guénard, 2023**
–	Propodeal spines approx. as long as declivity. Head dorsum longitudinally rugose. Body color black or brownish black	**57**
57	Head dorsum mainly longitudinally rugose. Body color brownish black [Oriental Region: China: Taiwan (type locality)] (see Qian and Xu 2024: fig. 26)	***T. yanwan* Terayama, 2009**
–	Head dorsum longitudinally rugose, smooth on the longitudinal central strip. Body color black [Oriental Region: China: Sichuan, Yunnan] (see Qian and Xu 2024: fig. 25)	***T. lidong* Qian & Xu, 2024**
58	Propodeal spines approx. as long as declivity	**59**
–	Propodeal spines obviously longer than declivity	**64**
59	Head dorsum mainly longitudinally rugose	**60**
–	Head dorsum reticulate	**62**
60	Petiolar node approx. conical with narrowly rounded summit. Body color black [Oriental Region: China: Yunnan] (see Qian and Xu 2024: fig. 13)	***T. daxue* Qian & Xu, 2024**
–	Petiolar node approx. triangular with bluntly angled summit. Body color yellowish brown	**61**
61	Head dorsum coarsely longitudinally rugose. Mesosomal dorsum weakly convex in lateral view [Palearctic Region: China: Qinghai; Russia (type locality)] (see Qian and Xu 2024: fig. 59)	***T. volgensis* (Ruzsky, 1905)**
–	Head dorsum finely longitudinally rugose. Mesosomal dorsum moderately convex in lateral view [Palearctic and Oriental Regions: China: Beijing, Gansu, Inner Mongolia, Jilin, Ningxia, Qinghai, Shaanxi, Yunnan] (see Qian and Xu 2024: fig. 60)	***T. reticulatus* (Chang & He, 2001)**
62	Posterior margin of head weakly convex. Mesosomal dorsum weakly convex [Palearctic Region: China: Jiangsu; North Korea] (see Qian and Xu 2024: fig. 57)	***T. xanthos* Radchenko, 2004**
–	Posterior margin of head nearly straight. Mesosomal dorsum moderately convex	**63**
63	Head dorsum and mesosoma densely reticulate [Oriental Region: China: Guangxi, Henan, Zhejiang] (see Qian and Xu 2024: fig. 56)	***T. zhejiangensis* Zhou et al., 2010**
–	Head dorsum and mesosoma loosely reticulate [Oriental Region: China: Guizhou, Hunan, Zhejiang] (see Qian and Xu 2024: fig. 58)	***T. ruginosus* Zhou et al., 2010**
64	Head dorsum reticulate. Petiolar node approx. trapezoidal, with distinct dorsal margin [Oriental Region: China: Yunnan] (fig. 9)	***T. hanlu* Qian & Xu, 2024**
–	Head dorsum longitudinally rugose. Petiolar node approx. conical or triangular, with narrowly rounded or bluntly angled summit	**65**
65	Head dorsum longitudinally rugose, smooth on the longitudinal central strip. Propodeal spines weakly up-curved. Petiolar node approx. conical, with narrowly rounded summit [Palearctic and Oriental Regions: China: Jiangxi (type locality); North Korea] (fig. 8)	***T. eburneipes* (Wheeler, 1927)**
–	Head dorsum wholly longitudinally rugose. Propodeal spines weakly down-curved. Petiolar node approx. triangular, with bluntly angled summit [Oriental Region: China: Hubei] (fig. 7)	***T. hubeiensis* sp. nov**.

### Descriptions of new species

#### Temnothorax shennongi sp. nov.

Taxon classificationAnimaliaHymenopteraFormicidae

A3FA4693-D96F-5196-B41C-7399350F1EA8

https://zoobank.org/A8FF95AC-54BE-450F-B902-08A27DC3021E

Fig. 1

##### Type material.

***Holotype***: • worker, China: Hubei Province, Badong County, Yanhedu Town, Xiaoshennongjia Village, 30°18'24"N, 110°21'34"E, 1150 m, foraging on the ground in semi-evergreen broadleaf forest, 20 June 2023, Fangchao Deng leg., SWFU A23-986. ***Paratype***: • 1 worker, China: Hubei Province, Shiyan City, Zhushan County, Mochi Village, 31°57'29"N, 110°04'40"E, 920 m, foraging on plant in semi-evergreen broadleaf forest, 9 June 2023, Fangchao Deng leg., SWFU A23-475.

**Figure 1.**
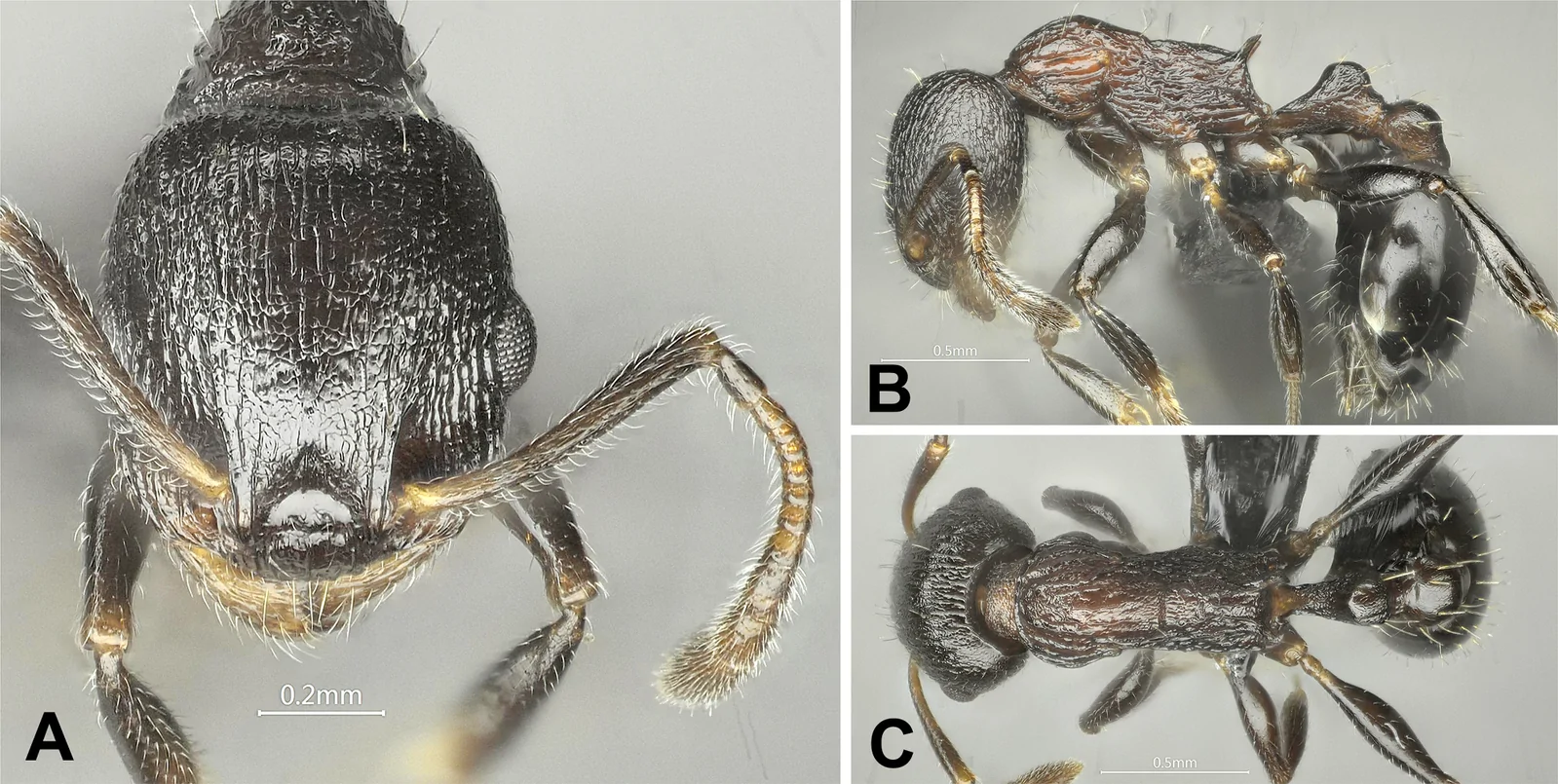
*Temnothorax shennongi* sp. nov. holotype worker (photographer: Zhenghui Xu). **A**. Head in full-face view; **B**. Body in lateral view; **C**. Body in dorsal view.

##### Description.

**Holotype worker** (Fig. 1): TL 3.2, HL 0.78, HW 0.68, CI 87, SL 0.58, SI 85, ED 0.18, PW 0.48, WL 0.98, PL 0.40, PH 0.25, DPW 0.20.

In full-face view head subrectangular, longer than broad, posterior margin slightly concave, posterior corners narrowly rounded, lateral margins weakly convex. Mandibles triangular, masticatory margin with five teeth. Clypeal dorsum weakly convex, with median carina, anterior margin moderately convex. Frontal lobes broad, concealing antennal sockets. Frontal carinae short, reaching to the level of midpoint of eyes. Antennae 12-segmented, scape failed to reach posterior head margin by 1/10 of its length, club 3-segmented. Eyes located at midline of head, occupying 1/4 of lateral margin.

In lateral view promesonotum strongly convex, promesonotal suture absent. Pronotum slightly convex, mesonotum almost straight and sloping down posteriorly, metanotal groove very shallowly impressed. Propodeal dorsum straight; propodeal spines moderately long and acute, posteriorly curved, ~ 2/3 length of declivity; declivity slightly concave, ~ 1/2 length of dorsum; propodeal lobes small and rounded apically. Petiole with relatively shorter anterior peduncle, ~ 0.7× as long as petiolar node; petiolar node approx. trapezoidal, anterior margin weakly concave, dorsal and posterior margins weakly convex, anterodorsal and posterodorsal corners narrowly rounded; ventral margin almost straight anteriorly and weakly concave posteriorly, anteroventral corner acutely toothed. Postpetiole approx. as high as petiole, dorsum roundly convex; ventral margin short, shallowly notched anteriorly and straight posteriorly.

In dorsal view pronotum broadest, anterior margin moderately convex, humeral corners broadly rounded, lateral margins strongly convex, promesonotal suture absent. Mesothorax moderately constricted, lateral margins moderately concave, metanotal groove very shallowly impressed. Propodeum approx. rectangular, lateral margins weakly convex; spines moderately long and pointed posterolaterally. Anterior peduncle of petiole constricted anteriorly, gradually widening posteriorly; petiolar node nearly square, slightly broader than long, anterior margin moderately convex, lateral and posterior margins slightly convex. Postpetiole approx. trapezoidal and narrowing posteriorly, ~ 1.4× as broad as petiole, anterolateral corners bluntly angled, lateral margins nearly straight. Gaster elongate oval, sting extruding.

Mandibles longitudinally striate. Head dorsum longitudinally rugose, rugae partially interwoven laterally. Clypeus loosely longitudinally rugose. Promesonotum loosely, coarsely and longitudinally rugose; mesopleura, metapleura and propodeum longitudinally undulate and rugose, rugae partially interwoven. Petiole and postpetiole finely densely punctate, petiole sides very loosely finely rugose, anterior face of petiolar node and anterior face of postpetiole smooth. Gaster smooth and shiny. Body dorsum with abundant erect to suberect apically blunt short hairs and abundant decumbent pubescence, hairs on mesosoma sparse. Scapes with dense subdecumbent pubescence, tibiae with dense decumbent pubescence. Body color dark reddish brown; femora, tibiae, and gaster black; mandibles and tarsi brownish yellow.

**Paratype worker**: TL 2.8, HL 0.65, HW 0.58, CI 88, SL 0.50, SI 87, ED 0.15, PW 0.43, WL 0.85, PL 0.33, PH 0.23, DPW 0.15 (*n* = 1). Resembling the holotype worker, but body relatively smaller, dorsum of petiolar node gently sloping down posteriorly, hairs on gaster sparse, dorsum of postpetiole smooth, body color yellowish brown, head blackish brown.

**Queen and male**. Unknown.

##### Ecological notes.

This new species inhabits semi-evergreen broadleaf forest with altitude 920–1150 m and forages on the ground and plants.

##### Distribution.

China: Hubei.

##### Comparative notes.

**Figure 2.**
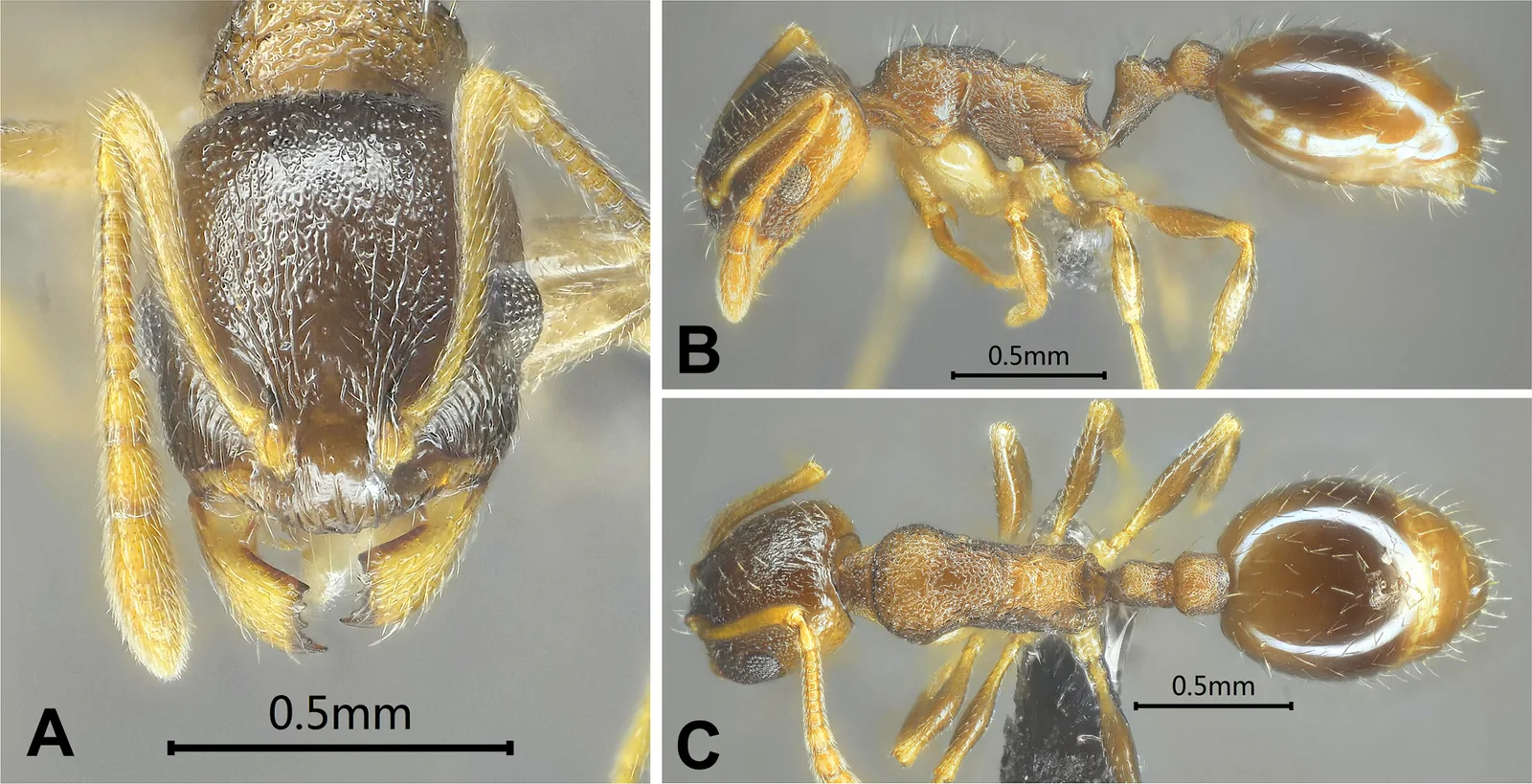
*Temnothorax liqiu* holotype worker (SWFU, images cited from Qian and Xu 2024: fig. 27). **A**. Head in full-face view; **B**. Body in lateral view; **C**. Body in dorsal view.

According to the species grouping and identification key, the new species is most similar to *Temnothorax liqiu* Qian & Xu, 2024 (Fig. 2), but differs in several characters. In the new species, promesonotum is strongly convex in lateral view; propodeal spines are relatively longer, ~ 1/2 the length of declivity; petiolar node is erect, with posterodorsal corner as high as anterodorsal corner; head dorsum and mesosoma are mainly longitudinally rugose. In contrast, in *T. liqiu*, promesonotum is weakly convex; propodeal spines are relatively shorter, ~ 1/4 length of declivity; petiolar node is weakly inclined posteriorly, with posterodorsal corner lower than anterodorsal corner; head dorsum and mesosoma are finely reticulate.

**Figure 3.**
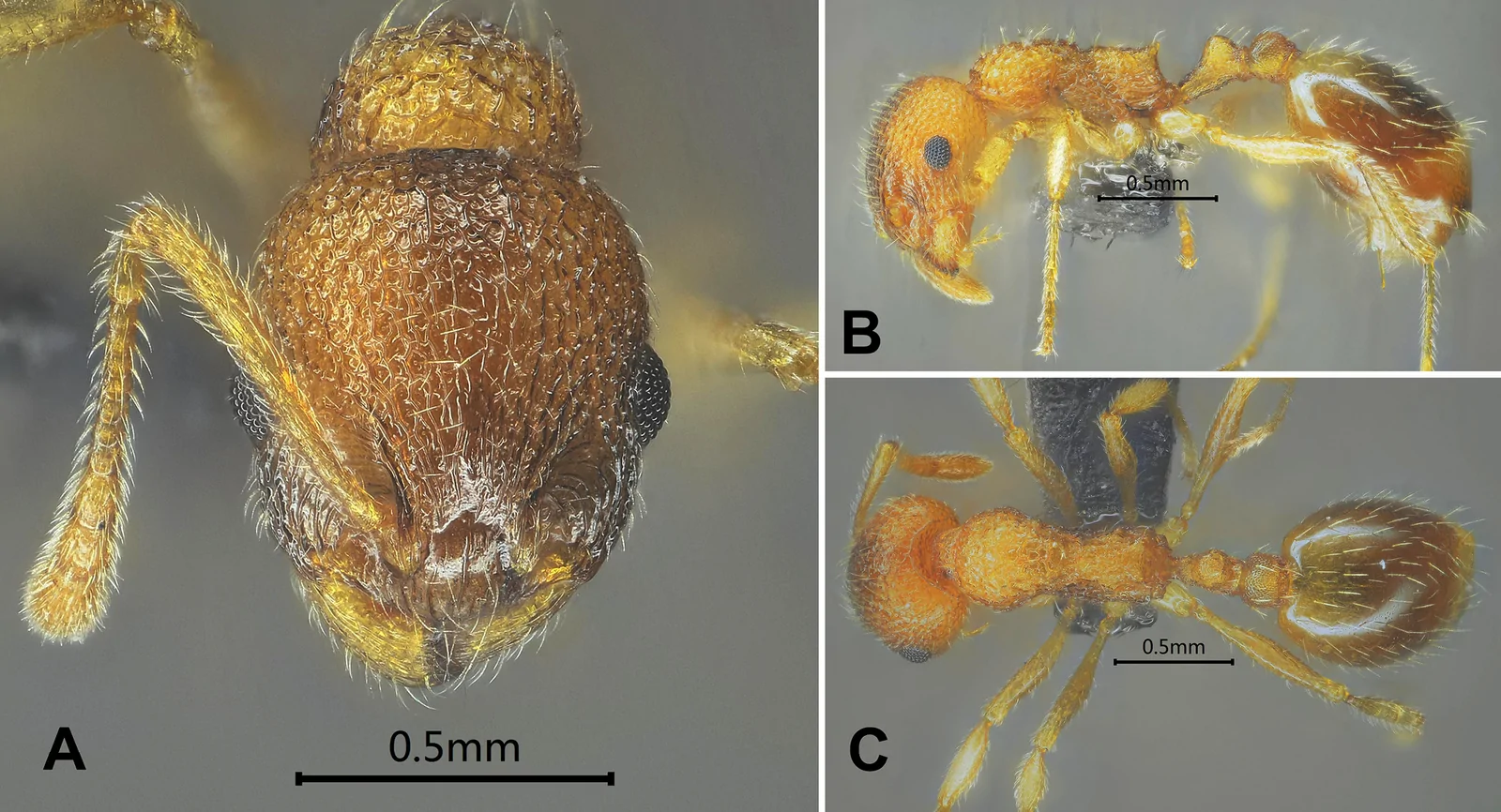
*Temnothorax xiaoshu* holotype worker (SWFU, images cited from Qian and Xu 2024: fig. 38). **A**. Head in full-face view; **B**. Body in lateral view; **C**. Body in dorsal view.

The new species is also similar to *Temnothorax xiaoshu* Qian & Xu, 2024 (Fig. 3), but can be distinguished as follows. In the new species, promesonotum is strongly convex in lateral view; propodeal spines are relatively longer, ~ 1/2 the length of declivity; petiolar node is approx. trapezoidal with broadly rounded summit; head dorsum and promesonotum are mainly longitudinally rugose. In *T. xiaoshu*, promesonotum is weakly convex; propodeal spines are relatively shorter, ~ 1/3 the length of declivity; petiolar node is approx. conical with narrowly rounded summit; head dorsum and promesonotum are coarsely reticulate.

##### Etymology.

The new species is named after “Shennong”, a legendary Emperor Yan, the Chinese god of agriculture and medicine. The type locality “Shennongjia” was also named after him.

#### Temnothorax wangweii sp. nov.

Taxon classificationAnimaliaHymenopteraFormicidae

EFB76FC1-80E7-5421-AC75-8D5746A47127

https://zoobank.org/791B6B4C-AA06-46BC-AC73-C2DF43BF92D8

Fig. 4

##### Type material.

***Holotype***: • worker, China: Hubei Province, Badong County, Yanhedu Town, Xiaoshennongjia Village, 30°18'29"N, 110°21'56"E, 700 m, foraging on the ground in semi-evergreen broadleaf forest, 19 June 2023, Fangchao Deng leg.; SWFU A23-906a.

**Figure 4.**
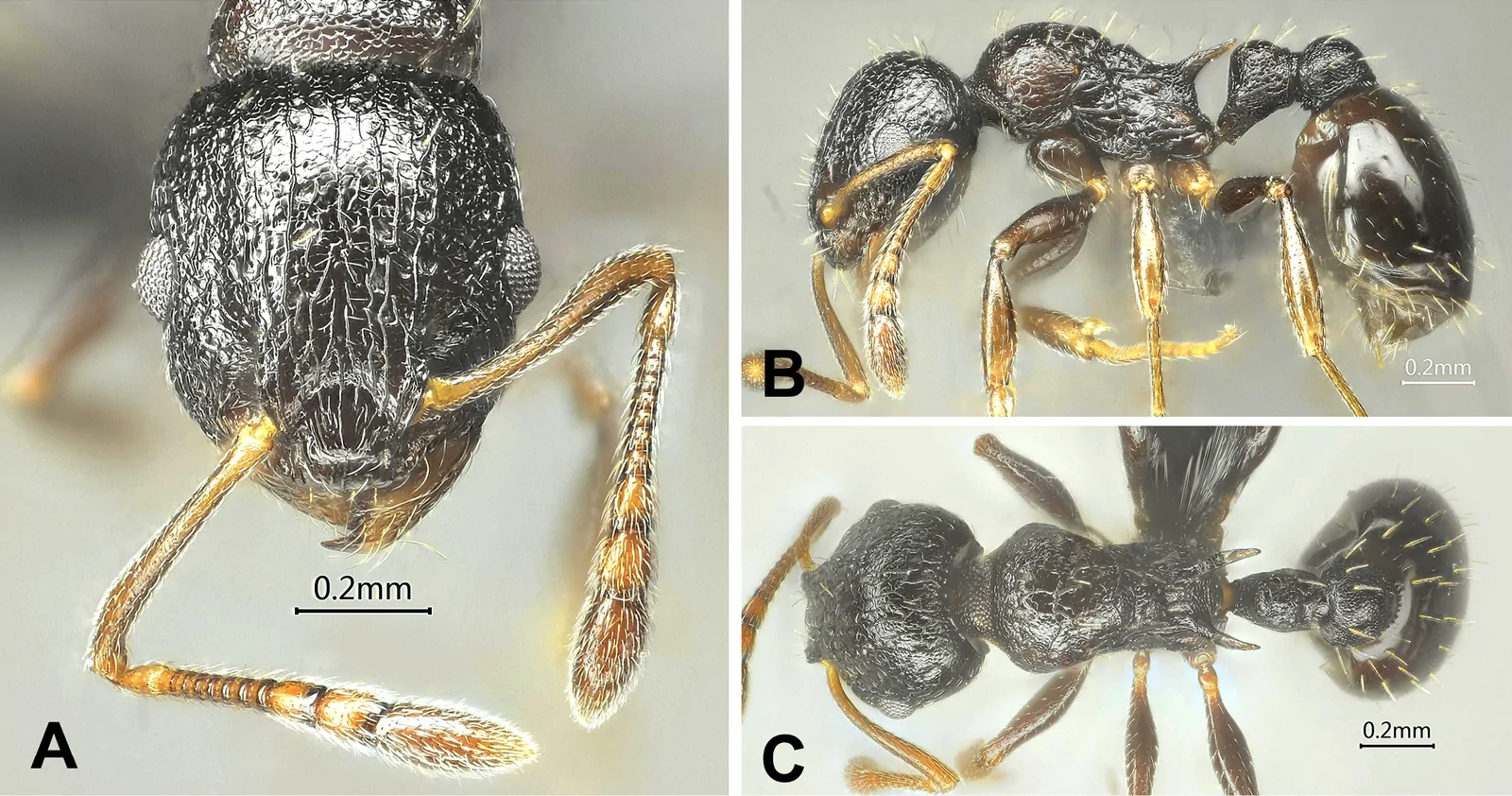
*Temnothorax wangweii* sp. nov. holotype worker (photographer: Zhenghui Xu). **A**. Head in full-face view; **B**. Body in lateral view; **C**. Body in dorsal view.

##### Description.

**Holotype worker** (Fig. 4): TL 2.4, HL 0.68, HW 0.58, CI 85, SL 0.50, SI 86, ED 0.16, PW 0.42, WL 0.74, PL 0.28, PH 0.22, DPW 0.18.

In full-face view head subrectangular, longer than broad, posterior margin straight, posterior corners narrowly rounded, lateral margins weakly convex. Mandibles triangular, masticatory margin with five teeth. Clypeal dorsum weakly convex, with fine median carina, anterior margin moderately convex. Frontal lobes broad, concealing antennal sockets. Frontal carinae long, surpassing the level of posterior margins of eyes. Antennae 12-segmented, scapes just reaching to posterior head margin, club 3-segmented. Eyes located at midline of head, occupying 1/4 of lateral margin.

In lateral view promesonotum high, pronotum moderately convex; mesonotum straight anteriorly, moderately convex, and sloping down posteriorly; promesonotal suture absent, metanotal groove moderately angularly impressed. Propodeal dorsum deeply concave and steeply sloping down posteriorly; propodeal spines long, thick at base and thin at end, weakly down-curved, ~ 1.4× as long as declivity; declivity weakly concave, shorter than dorsum; propodeal lobes small and rounded apically. Petiole with short, thin, anterior peduncle, ~ 1/2 length of petiolar node; petiolar node approx. conical, anterior margin nearly straight, ~ 1/2 length of posterior margin; summit narrowly rounded, posterior margin weakly convex; ventral margin slightly concave, anteroventral corner right-angled. Postpetiole ~ 1.1× as high as petiole, anterodorsal margin rounded, posterior margin nearly straight; ventral margin very short and nearly straight.

In dorsal view pronotum broadest, anterior margin moderately convex, humeral corners narrowly rounded, lateral margins slightly convex. Mesothorax weakly constricted, lateral margins weakly concave. Promesonotal suture absent, metanotal groove moderately impressed. Propodeum approx. rectangular, broader than long, lateral margins weakly convex; spines long and moderately in-curved, apices acute. Petiole strongly constricted anteriorly, gradually widening posteriorly and approx. trapezoidal; petiolar node approx. circular, approx. as broad as long. Postpetiole approx. trapezoidal and narrowing posteriorly, ~ 1.4× as broad as petiole, anterior and lateral margins nearly straight, anterolateral corners bluntly angled, posterior margin moderately convex. Gaster elongate oval, sting extruding.

Mandibles longitudinally striate. Head dorsum loosely longitudinally rugose and partially connected by short transverse rugae, reticulate laterally. Clypeus loosely longitudinally rugose laterally. Mesosoma loosely reticulate; mesopleura loosely longitudinally rugose; petiole and postpetiole densely punctate, lateral margins loosely reticulate. Gaster smooth and shiny. Body dorsum with abundant erect to subdecumbent apically blunt short hairs and abundant decumbent pubescence. Scapes and tibiae with dense depressed pubescence. Body color black; pronotum and gaster blackish brown; mandibles, antennae, and legs yellowish brown.

**Queen and male**. Unknown.

##### Ecological notes.

This new species inhabits semi-evergreen broadleaf forest with altitude 700 m and forages on the ground.

##### Distribution.

China: Hubei.

##### Comparative notes.

**Figure 5.**
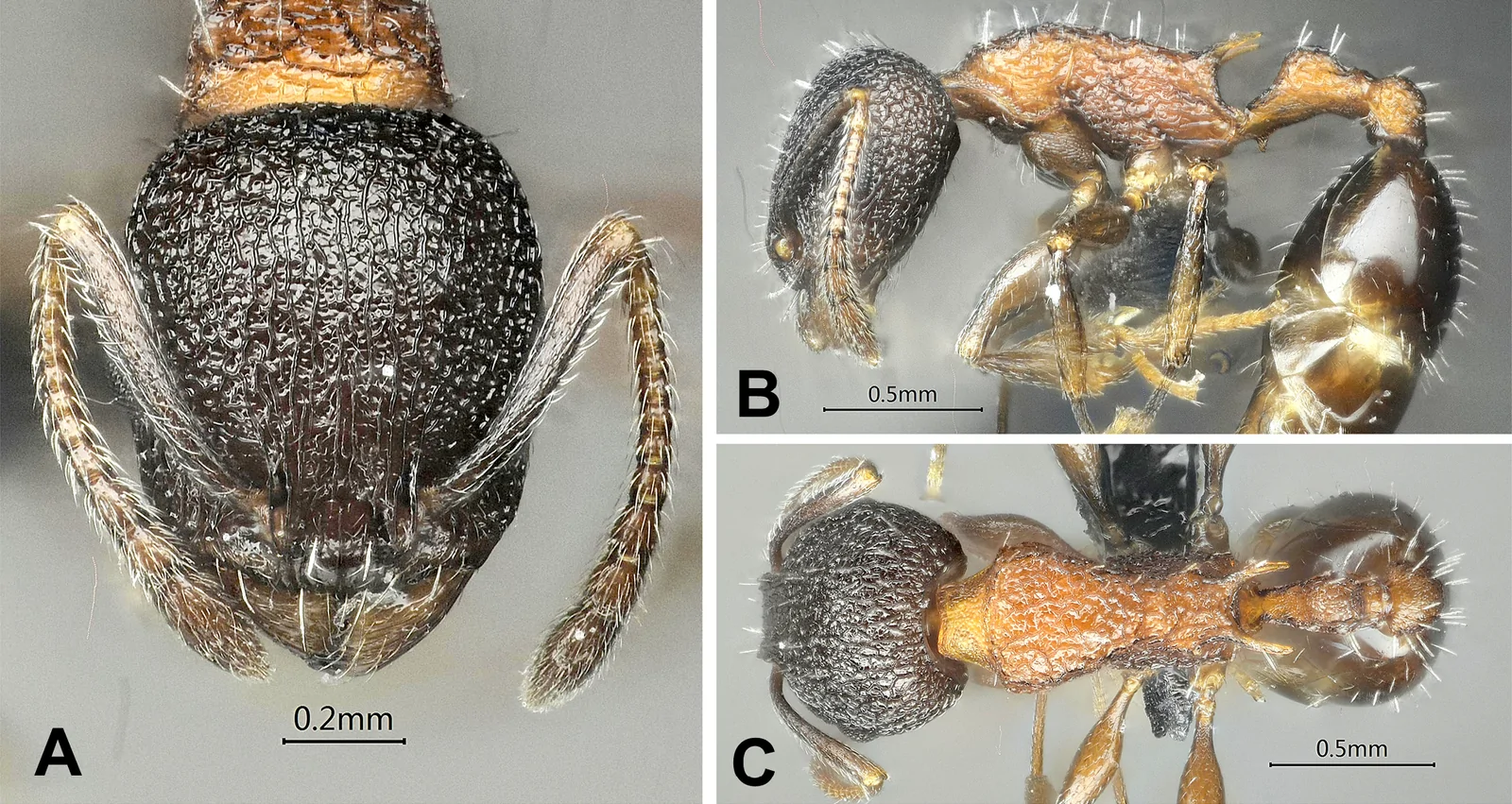
*Temnothorax lichun* holotype worker (SWFU, images cited from Qian and Xu, 2024: fig. 23). **A**. Head in full-face view; **B**. Body in lateral view; **C**. Body in dorsal view.

According to the species grouping and identification key, the new species is most similar to *Temnothorax lichun* Qian & Xu, 2024 (Fig. 5), but differs in several characters. In the new species, promesonotum is strongly convex in lateral view; metanotal groove is narrowly notched; anterior peduncle is ~ 1/2 the length of petiolar node, dorsum of the node is strongly convex; head dorsum is loosely longitudinally rugose. In contrast, in *T. lichun*, promesonotum is weakly convex in lateral view, metanotal groove is weakly impressed; anterior peduncle is ~ 2/3 the length of petiolar node, dorsum of the node is weakly convex; head dorsum is densely reticulate.

**Figure 6.**
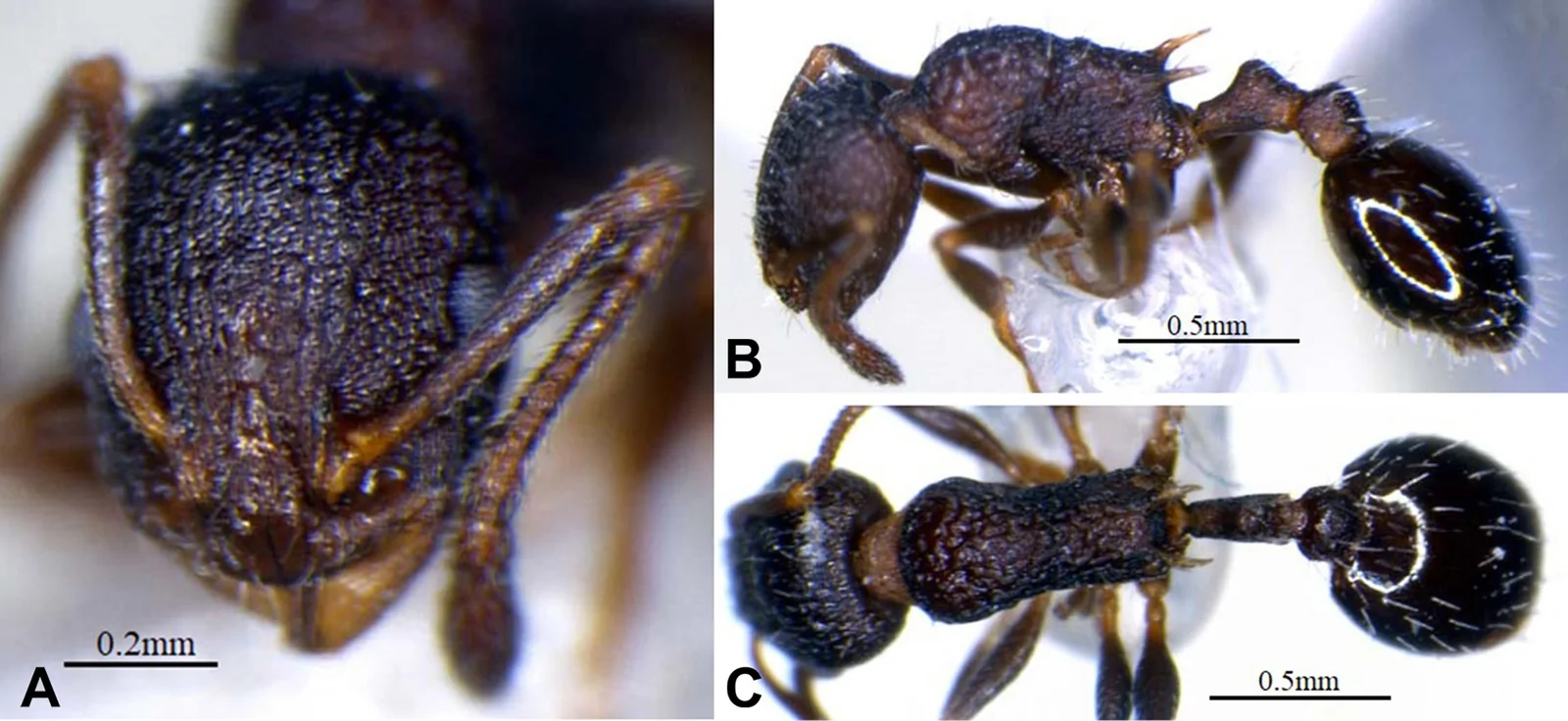
*Temnothorax kuixing* holotype worker (NIAS, images cited from www.antwiki.org, photos by Hiraku Yoshitake & Takashi Kurihara). **A**. Head in full-face view; **B**. Body in lateral view; **C**. Body in dorsal view.

The new species is also similar to *Temnothorax kuixing* Terayama, 2009 (Fig. 6), but can be distinguished as follows. In the new species, metanotal groove is narrowly notched; petiolar node is approx. trapezoidal with broadly rounded dorsum, anterior peduncle is ~ 1/2 length of the node; head dorsum is loosely longitudinally rugose with interface smooth. In *T. kuixing*, metanotal groove is weakly impressed; petiolar node is approx. conical with narrowly rounded dorsum, anterior peduncle is ~ 2/3 length of the node; head dorsum is finely reticulate with interface punctate.

##### Etymology.

The new species is named after Dr. Wei Wang (Hubei Minzu University, Enshi), a Chinese myrmecologist who has made significant contributions to the study of ant fauna in Hubei Province of China.

#### Temnothorax hubeiensis sp. nov.

Taxon classificationAnimaliaHymenopteraFormicidae

CADFF8CE-AF01-53F0-836F-C6369A932909

https://zoobank.org/8A8CE1FD-FD18-4014-A510-363349B24651

Fig. 7

##### Type material.

***Holotype***: • worker, China: Hubei Province, Badong County, Yanhedu Town, Xiaoshennongjia Village, 30°18'29"N, 110°21'56"E, 550 m, foraging on the ground in semi-evergreen broadleaf forest, 19 June 2023, Fangchao Deng leg., SWFU A23-864. ***Paratypes***: • 1 worker, data the same as holotype, but 570 m, SWFU A23-848; • 1 worker, data the same as holotype, but 600 m, SWFU A23-889; • 1 worker, data the same as holotype, but 700 m, SWFU A23-906, the paratype specimen will be donated to the Insect Collection, Guangxi Normal University, Guilin, Guangxi Zhuang Autonomous Region, China.

**Figure 7.**
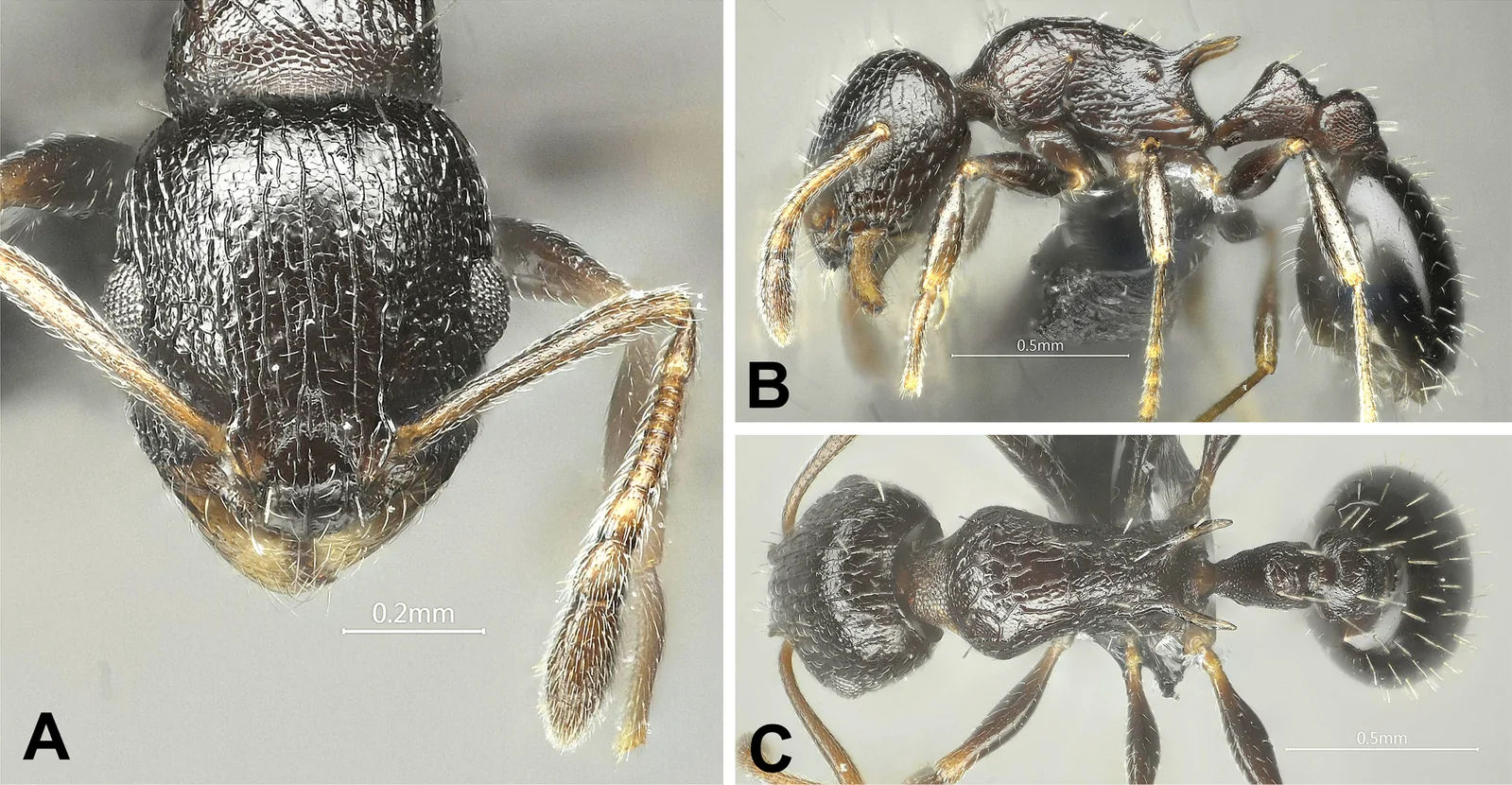
*Temnothorax hubeiensis* sp. nov. holotype worker (photographer: Zhenghui Xu). **A**. Head in full-face view; **B**. Body in lateral view; **C**. Body in dorsal view.

##### Description.

**Holotype worker** (Fig. 7): TL 2.5, HL 0.64, HW 0.54, CI 84, SL 0.50, SI 93, ED 0.16, PW 0.42, WL 0.70, PL 0.28, PH 0.22, DPW 0.18.

In full-face view head subrectangular, longer than broad, posterior margin slightly concave in the center, posterior corners narrowly rounded, lateral margins weakly convex. Mandibles triangular, masticatory margin with five teeth. Clypeal dorsum weakly convex, with median carina, anterior margin moderately convex. Frontal lobes broad, concealing antennal sockets. Frontal carinae short, reaching to the level of midpoint of eyes. Antennae 12-segmented, scapes just reaching to posterior head margin, club 3-segmented. Eyes located at midline of head, occupying 1/4 of lateral margin.

In lateral view mesosomal dorsum weakly convex and weakly arched, pronotum moderately convex, promesonotal suture and metanotal groove absent. Propodeal dorsum slightly convex and sloping down posteriorly; propodeal spines long and slender, down-curved at base, the apical 2/3 straight, ~ 1.3× as long declivity; declivity weakly concave, shorter than dorsum; propodeal lobes small and rounded apically. Petiole with short anterior peduncle, ~ 1/2 length of petiolar node; petiolar node approx. triangular, anterior and posterior margins nearly straight, summit bluntly angled; ventral margin almost straight anteriorly and weakly concave posteriorly, anteroventral corner minutely toothed. Postpetiole approx. as high as petiole, weakly inclining anteriorly, anterior margin short and nearly straight, anterodorsal corner narrowly rounded, dorsum weakly convex and sloping down posteriorly; ventral margin short, shallowly concave.

In dorsal view pronotum broadest, anterior margin moderately convex, humeral corners narrowly rounded, lateral margins of mesothorax weakly concave, promesonotal suture and metanotal groove absent. Propodeum approx. rectangular, lateral margins nearly straight; spines long and pointed posterolaterally, weakly in-curved. Anterior peduncle of petiole strongly constricted anteriorly, gradually widening posteriorly; petiolar node approx. trapezoidal and weakly widening posteriorly, lateral and posterior margins slightly convex. Postpetiole approx. trapezoidal and narrowing posteriorly, ~ 1.4× as broad as petiole, anterolateral corners narrowly rounded, lateral margins nearly straight. Gaster elongate oval, sting present.

Mandibles longitudinally striate. Head dorsum loosely longitudinally rugose, reticulate laterally. Clypeus loosely longitudinally rugose laterally. Mesosoma loosely reticulate; mesonotum, mesopleura, and metapleura loosely longitudinally rugose; petiole and postpetiole loosely reticulate and finely punctate, anterior face of petiolar node and anterior face of postpetiole relatively smooth. Gaster smooth and shiny. Body dorsum with abundant erect to suberect apically blunt short hairs and abundant decumbent pubescence, hairs on mesosoma sparse. Scapes and tibiae with dense decumbent to depressed pubescence. Body color dark reddish brown; mandibles pale yellow; antennae and legs blackish brown; gaster black.

**Paratype workers**: TL 2.5–2.7, HL 0.62–0.68, HW 0.52–0.58, CI 82–85, SL 0.48–0.50, SI 86–92, ED 0.13–0.16, PW 0.38–0.42, WL 0.70–0.76, PL 0.26–0.30, PH 0.22–0.24, DPW 0.16–0.19 (*n* = 3). Similar to holotype worker, but body relatively larger or smaller, summit of petiolar node bluntly angled or narrowly rounded, anterior face of petiolar node and anterior face of postpetiole smooth or finely punctate, antennae and legs blackish brown or yellowish brown.

**Queen and male**. Unknown.

##### Ecological notes.

This new species inhabits semi-evergreen broadleaf forest with altitude 550–700 m and forages on the ground.

##### Distribution.

China: Hubei.

##### Comparative notes.

**Figure 8.**
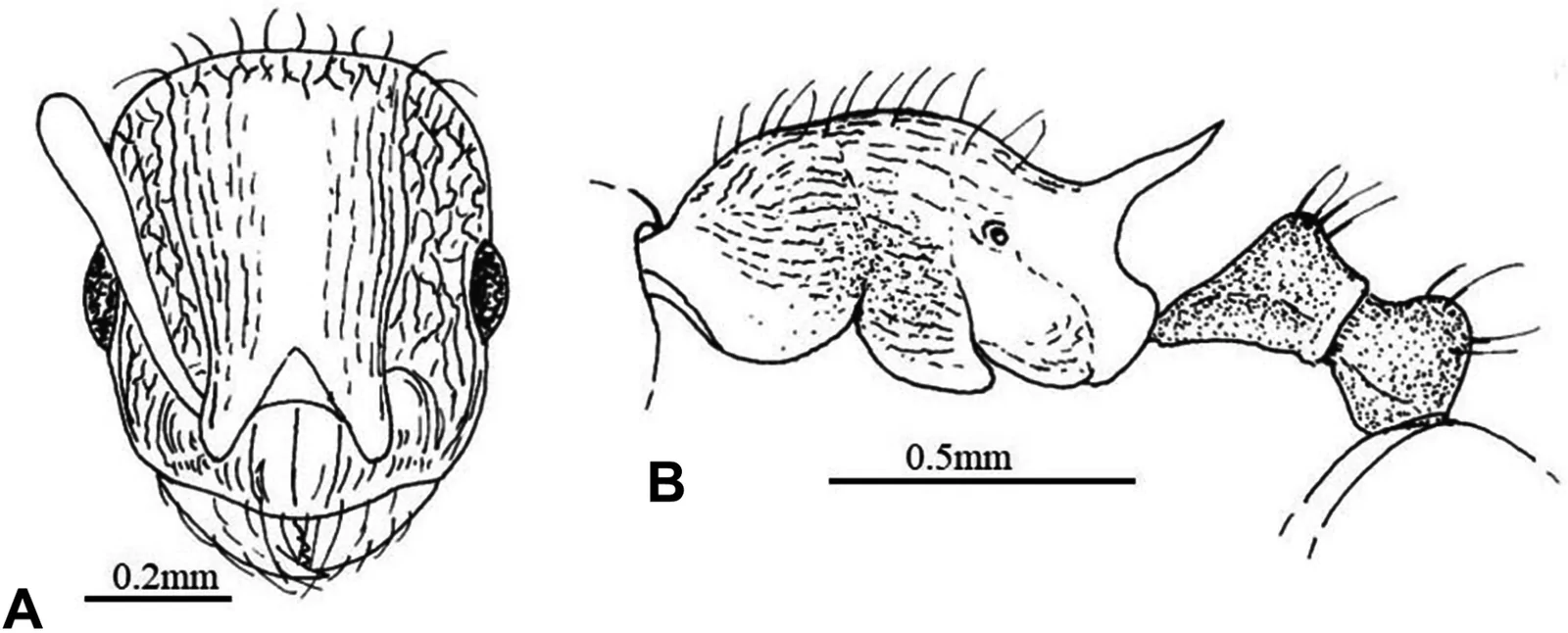
*Temnothorax eburneipes* syntype worker (MCZC, illustrations cited from Radchenko 2004). **A**. Head in full-face view; **B**. Body in lateral view.

According to the species grouping and identification key, the new species is most similar to *Temnothorax eburneipes* (Wheeler, 1927) (Fig. 8) but differs in several characters. In the new species, head dorsum is wholly longitudinally rugose; propodeal spines are weakly down-curved in lateral view; petiolar node is approx. triangular, with bluntly angled summit; body color black. In contrast, in *T. eburneipes*, head dorsum is longitudinally rugose and smooth on the longitudinal central strip; propodeal spines are weakly up-curved in lateral view; petiolar node is approx. conical, with narrowly rounded summit; body color blackish brown.

**Figure 9.**
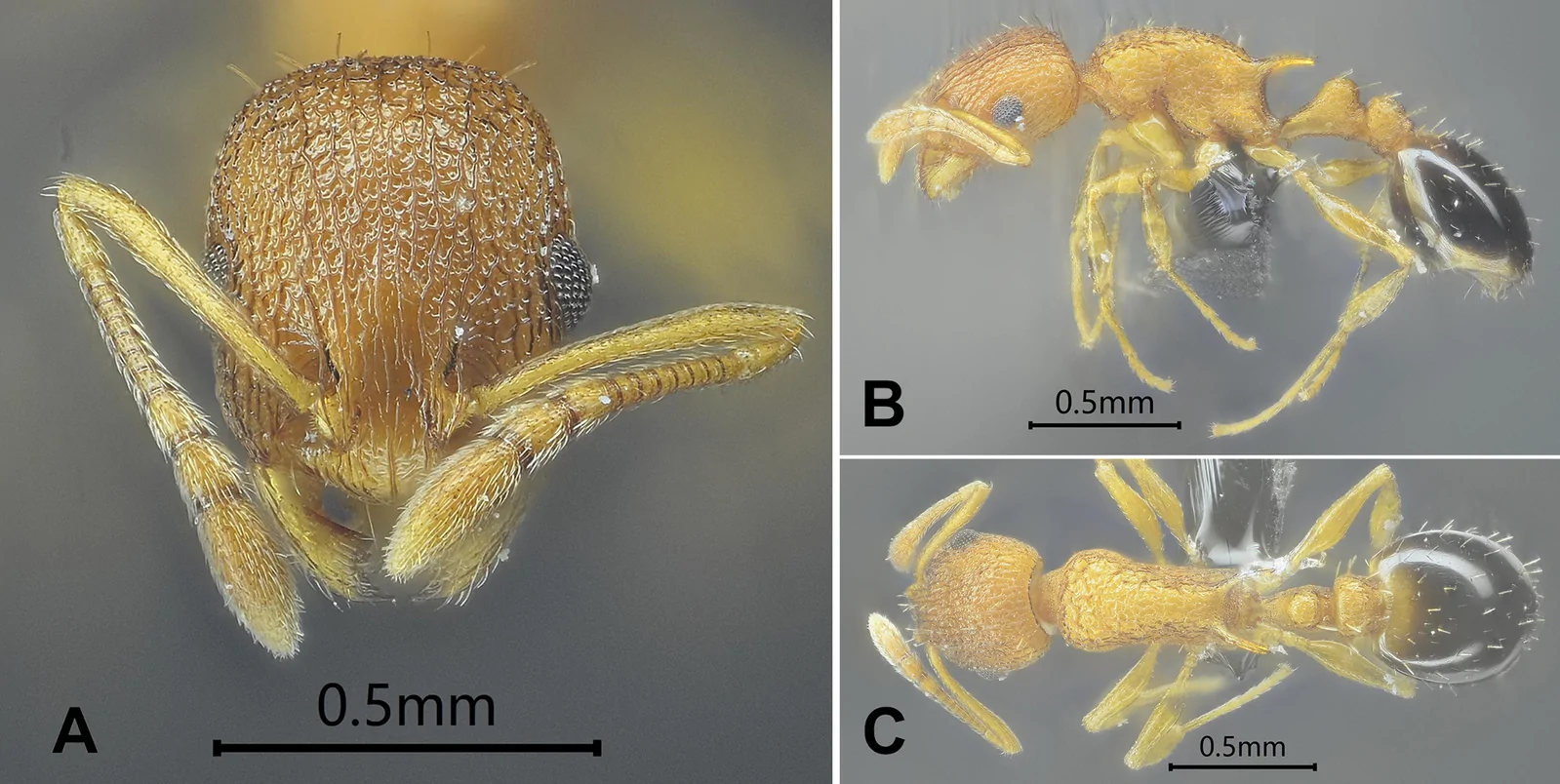
*Temnothorax hanlu* holotype worker (SWFU, images cited from Qian and Xu, 2024: fig. 21). **A**. Head in full-face view; **B**. Body in lateral view; **C**. Body in dorsal view.

The new species is also similar to *Temnothorax hanlu* Qian & Xu, 2024 (Fig. 9), but can be distinguished as follows. In the new species, propodeal spines are moderately curved backward at base; petiolar node is approx. triangular and erect, with bluntly angled summit; head and gaster are black, while mesosoma, petiole and postpetiole are blackish brown. In *T. hanlu*, propodeal spines are evenly curved backwards; petiolar node is approx. trapezoidal and inclining posteriorly, with broadly rounded summit; body color is brownish yellow, while gaster is black.

##### Etymology.

The new species is named after the type locality “Hubei Province”.

#### Temnothorax zhaojunae sp. nov.

Taxon classificationAnimaliaHymenopteraFormicidae

9E671E7D-4899-5E74-A1C0-762D8FC6E874

https://zoobank.org/04642665-8AF5-4315-8B68-56CCFD903205

Fig. 10

##### Type material.

***Holotype***: • worker, China: Hubei Province, Shennongjia Forestry District, Muyu Town, Qianjiaping Village, 31°24'43"N, 110°24'17"E, 1700 m, foraging on the plant in conifer-broadleaf mixed forest, 14 June 2023, Fangchao Deng leg., SWFU A23-743. ***Paratype***: • 1 worker, data the same as holotype, but SWFU A23-732.

**Figure 10.**
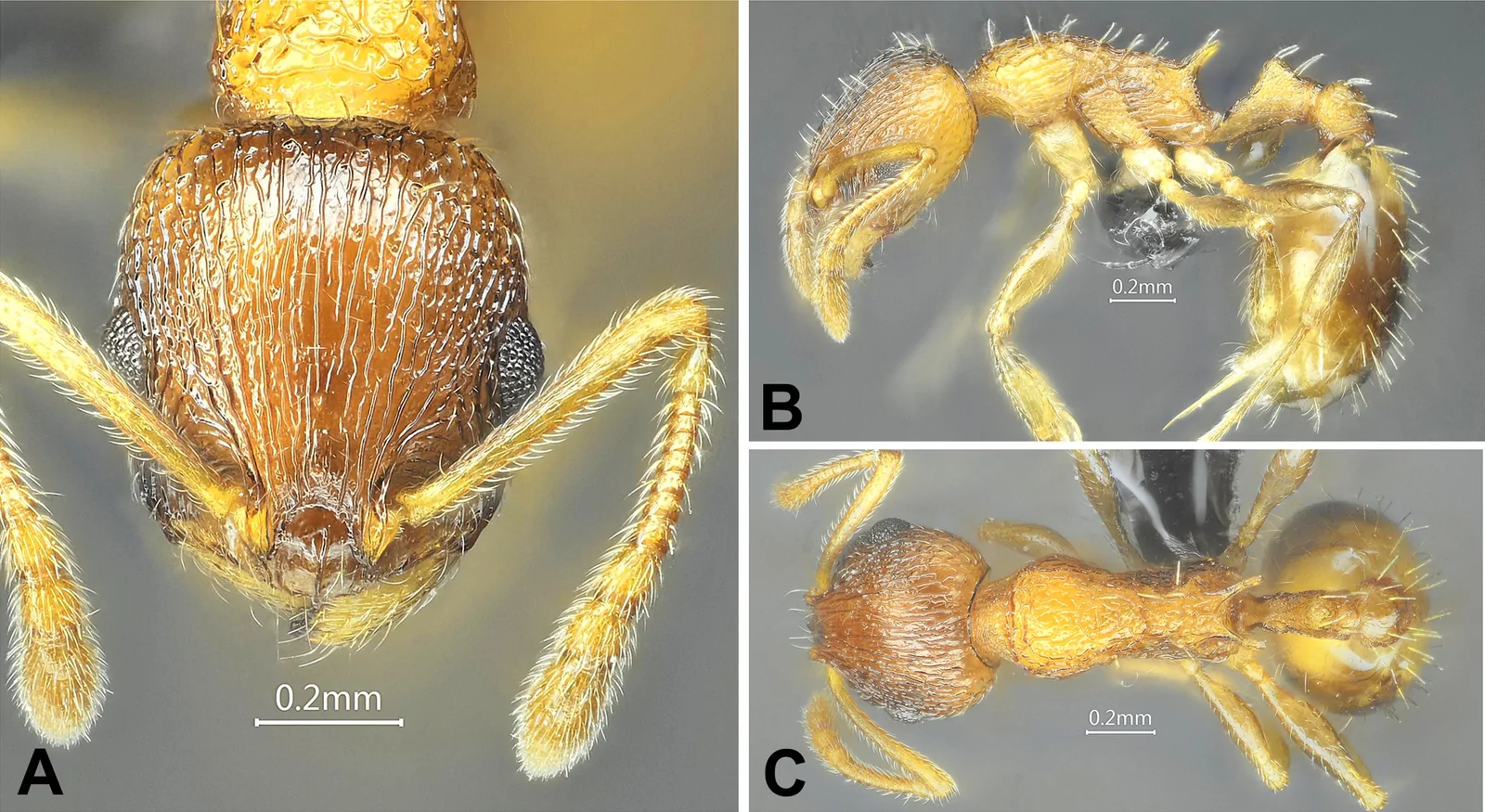
*Temnothorax zhaojunae* sp. nov. holotype worker (photographer: Zhenghui Xu). **A**. Head in full-face view; **B**. Body in lateral view; **C**. Body in dorsal view.

##### Description.

**Holotype worker** (Fig. 10): TL 2.7, HL 0.68, HW 0.56, CI 82, SL 0.52, SI 93, ED 0.16, PW 0.38, WL 0.80, PL 0.28, PH 0.22, DPW 0.14.

In full-face view head subrectangular, longer than broad, posterior margin straight, posterior corners narrowly rounded, lateral margins weakly convex. Mandibles triangular, masticatory margin with five teeth. Clypeal dorsum weakly convex, with median carina, anterior margin moderately convex. Frontal lobes broad, concealing antennal sockets. Frontal carinae very short, reaching to the level of anterior margins of eyes. Antennae 12-segmented, scapes just reaching to posterior head margin, club 3-segmented. Eyes located at midline of head, occupying 1/3 of lateral margin.

In lateral view promesonotum moderately convex and weakly arched, mesonotum gently sloping down posteriorly, promesonotal suture absent, metanotal groove weakly impressed. Propodeal dorsum straight, gently sloping down posteriorly; propodeal spines long and slender, weakly posteriorly curved, as long as declivity; declivity weakly concave, shorter than dorsum; propodeal lobes small and rounded apically. Petiole with short anterior peduncle, ~ 0.8× as long as petiolar node; petiolar node approx. triangular, anterior margin nearly straight, posterior margins weakly convex, summit right-angled; ventral margin almost straight, weakly concave posteriorly, anteroventral corner with a developed acute short spine, anteroventrally pointed. Postpetiole ~ 0.7× as high as petiole, weakly inclining anteriorly, anterior margin strongly convex, anterodorsal corner narrowly rounded, dorsum nearly straight and sloping down posteriorly; ventral margin short, deeply concave.

In dorsal view pronotum broadest, anterior margin moderately convex, humeral corners broadly rounded, lateral margins moderately convex. Mesothorax weakly constricted, lateral margins moderately concave. Promesonotal suture and metanotal groove absent. Propodeum approx. trapezoidal and widening posteriorly, lateral margins weakly concave; spines long and weakly in-curved, apices blunt. Petiole strongly constricted anteriorly, gradually widening posteriorly and approx. trapezoidal; petiolar node transverse and approx. elliptical, broader than long. Postpetiole approx. rectangular, broader than long, ~ 1.4× as broad as petiole, anterior margin weakly convex, anterolateral corners bluntly angled, lateral margins straight. Gaster elongate oval, sting extruding.

Mandibles longitudinally striate. Head dorsum abundantly longitudinally rugose, rugae weakly diverging posteriorly, and reticulate laterally. Clypeus loosely longitudinally rugose laterally. Mesosomal dorsum loosely reticulate; pronotal sides and metapleura longitudinally rugose, mesopleura reticulate; propodeal sides weakly reticulate; petiole and postpetiole densely punctate and finely reticulate, petiole side with a longitudinal ridge, anterior face of postpetiole relatively smooth. Gaster smooth and shiny. Body dorsum with abundant erect to subdecumbent apically blunt short hairs and abundant decumbent pubescence. Scapes with dense decumbent pubescence, tibiae with dense depressed pubescence. Body color brownish yellow, head dorsum reddish brown, legs and posterior 2/3 of gaster blackish brown.

**Paratype worker**: TL 2.7, HL 0.68, HW 0.54, CI 79, SL 0.52, SI 96, ED 0.16, PW 0.36, WL 0.80, PL 0.28, PH 0.22, DPW 0.14 (*n* = 1). Similar to holotype worker, but frontal carinae reaching to the level of midpoint of eyes, pronotal sides loosely reticulate, posterior margin of petiolar node moderately convex, legs brownish yellow.

**Queen and male**. Unknown.

##### Ecological notes.

This new species inhabits conifer-broadleaf mixed forest with altitude 1700 m and forages on the plants.

##### Distribution.

China: Hubei.

##### Comparative notes.

**Figure 11.**
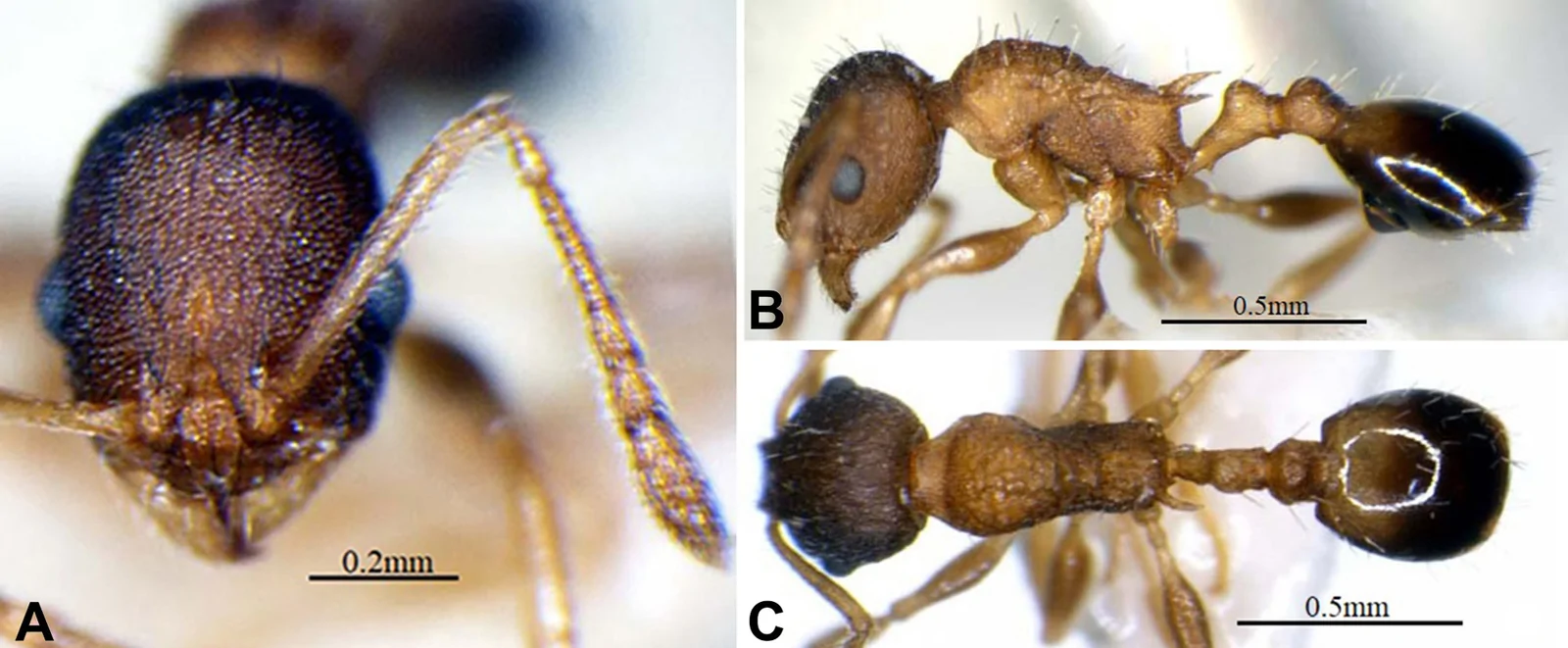
*Temnothorax leigong* holotype worker (NIAS, images cited from www.antwiki.org, photos by Hiraku Yoshitake & Takashi Kurihara). **A**. Head in full-face view; **B**. Body in lateral view; **C**. Body in dorsal view.

According to the species grouping and identification key, the new species is most similar to *Temnothorax leigong* Terayama, 2009 (Fig. 11), but differs in several characters. In the new species, head dorsum is longitudinally rugose with interface smooth; apex of scape is just reached posterior corner of head; petiolar node is approx. triangular and symmetrical, with right-angled summit, anterior margin is weakly concave. In contrast, in *T. leigong*, head dorsum is loosely longitudinally rugose with interface densely reticulate; apex of scape is well surpassed posterior corner of head; petiolar node is approx. triangular and asymmetrical, with bluntly angled summit, anterior margin is strongly concave.

The new species is also similar to *Temnothorax kuixing* Terayama, 2009 (Fig. 6), but can be distinguished as follows. In the new species, head dorsum is longitudinally rugose with interface smooth; apex of scape just reaches posterior corner of head; petiolar node is approx. triangular and symmetrical, with right-angled summit, anterior margin is weakly concave. In *T. kuixing*, head dorsum is longitudinally rugose with interface densely reticulate; apex of scape clearly surpasses posterior corner of head; petiolar node is approx. conical and inclined anteriorly, with narrowly rounded summit, anterior margin is strongly concave.

##### Etymology.

The new species is named after “Zhaojun Wang”, one of the four great beauties in ancient China. She married into the Huns in the Han Dynasty for the purpose of peace. Her hometown was Hubei, the type locality.

## Supplementary Material

XML Treatment for Temnothorax shennongi

XML Treatment for Temnothorax wangweii

XML Treatment for Temnothorax hubeiensis

XML Treatment for Temnothorax zhaojunae

## Additional information

### Artificial Intelligence (AI) use

The authors accept full responsibility for the content of the manuscript, including the disclosure of any use of AI.

No AI tools were used in the preparation of this manuscript.

### Author ORCIDs

F. Deng https://orcid.org/0009-0000-1595-6361

J. Yang https://orcid.org/0000-0003-4861-8267

Z. Xu https://orcid.org/0000-0003-2415-770X

### Data availability

All of the data that support the findings of this study are available in the main text.
